# Low RNA Polymerase III activity results in up regulation of *HXT2* glucose transporter independently of glucose signaling and despite changing environment

**DOI:** 10.1371/journal.pone.0185516

**Published:** 2017-09-29

**Authors:** Malgorzata Adamczyk, Roza Szatkowska

**Affiliations:** Institute of Biotechnology, Faculty of Chemistry, Warsaw University of Technology, Warsaw, Poland; University of Szeged, HUNGARY

## Abstract

**Background:**

*Saccharomyces cerevisiae* responds to glucose availability in the environment, inducing the expression of the low-affinity transporters and high-affinity transporters in a concentration dependent manner. This cellular decision making is controlled through finely tuned communication between multiple glucose sensing pathways including the Snf1-Mig1, Snf3/Rgt2-Rgt1 (SRR) and cAMP-PKA pathways.

**Results:**

We demonstrate the first evidence that RNA Polymerase III (RNAP III) activity affects the expression of the glucose transporter *HXT2* (RNA Polymerase II dependent—RNAP II) at the level of transcription. Down-regulation of RNAP III activity in an *rpc128-1007* mutant results in a significant increase in *HXT2* mRNA, which is considered to respond only to low extracellular glucose concentrations. *HXT2* expression is induced in the mutant regardless of the growth conditions either at high glucose concentration or in the presence of a non-fermentable carbon source such as glycerol. Using chromatin immunoprecipitation (ChIP), we found an increased association of Rgt1 and Tup1 transcription factors with the highly activated *HXT2* promoter in the *rpc128-1007* strain. Furthermore, by measuring cellular abundance of Mth1 corepressor, we found that in *rpc128-1007*, *HXT2* gene expression was independent from Snf3/Rgt2-Rgt1 (SRR) signaling. The Snf1 protein kinase complex, which needs to be active for the release from glucose repression, also did not appear perturbed in the mutated strain.

**Conclusions/Significance:**

These findings suggest that the general activity of RNAP III can indirectly affect the RNAP II transcriptional machinery on the *HXT2* promoter when cellular perception transduced *via* the major signaling pathways, broadly recognized as on/off switch essential to either positive or negative *HXT* gene regulation, remain entirely intact. Further, Rgt1/Ssn6-Tup1 complex, which has a dual function in gene transcription as a repressor-activator complex, contributes to *HXT2* transcriptional activation.

## Introduction

Elucidation of glucose signaling mechanisms and gene regulation in eukaryotic cells as well as its impact on cellular processes is a fundamental issue for the efficient treatment of metabolic disorders such as cancer and diabetes. Additionally, when studied in yeast cells, it facilities the intelligent design and engineering of the microorganism for the improvement of industrial processes, such as the conversion of biological substances to industrially interesting products of high value [1], [2].

To sustain growth and development, *S*. *cerevisiae* ensures the optimal utilization of glucose by adjusting its transcriptional program to the availability of the key metabolite [3]. It is a wildly accepted knowledge that presence of glucose, in the external environment is signaled to the related genes. This signal transduction is performed by a cascade of proteins, that communicate the signal *via* phosphorylation to transcription factors and chromatin remodeling complexes [4]. Glucose signaling represses a large set of yeast genes to achieve reprogramming of the cell metabolism in response to availability of glucose and glucose concentrations. For instance the reprogramming is observed during the transition from fermentative growth on glucose and the phase of oxidative phosphorylation when glucose starts depleting. Consequently, the cells require the activity of enzymes for the metabolism of non-fermentable carbon sources (Fbp1, Pck1, Pyk2 and many others). Long term glucose repression and glucose induction of gene expression on the genomic scale, act mainly at the transcriptional level [5], [6].

Signaling encompasses the entire process of sensing stimuli, generating intracellular signals, signal transduction and the generation of an appropriate response.

Expression of the major group of glucose transporters, *HXT1-4* and *HXT6* and *HXT7*, is mainly controlled by the Snf3/Rgt2-Rgt1 (SRR) signaling pathway [7]. However, additional pathways are involved in expression of these transporters, such as the Snf1-Mig1 and the cAMP-PKA pathway [8], [3], [9], [10].

**Fig 1:**
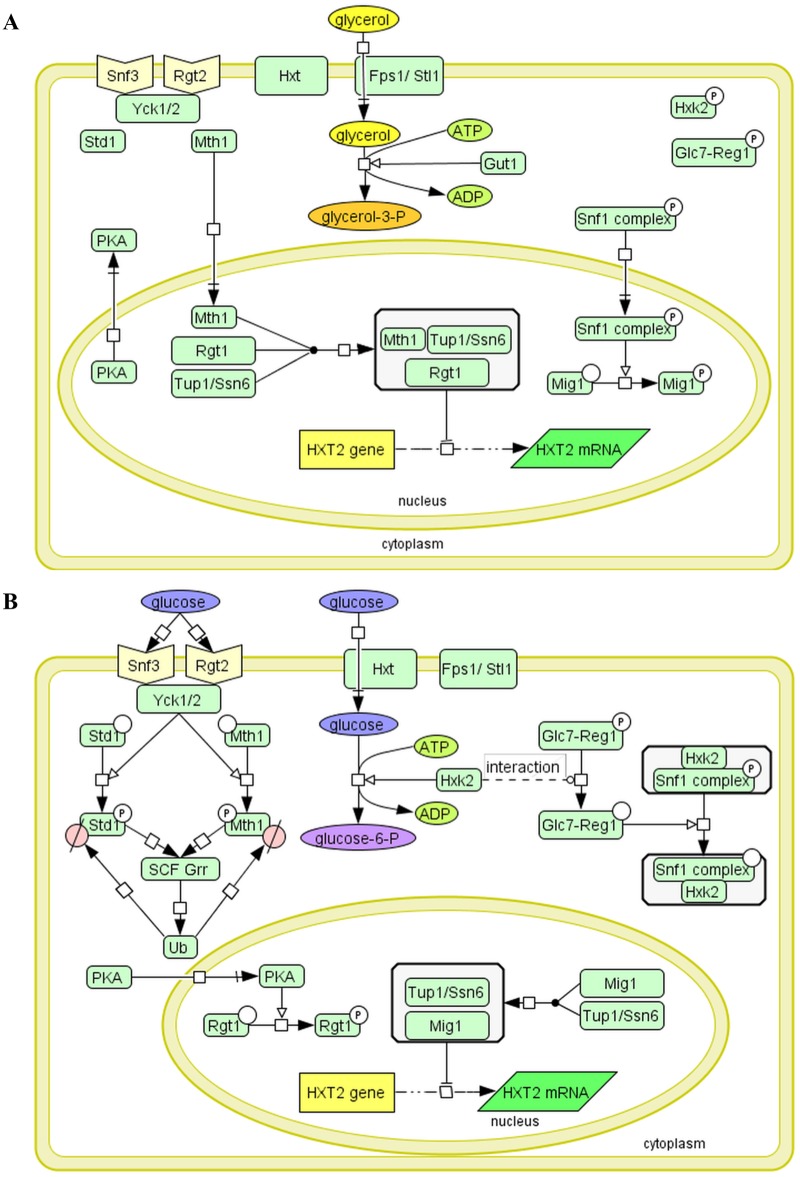
Diagram illustrating the crosstalk between the Snf1-Mig1 and Snf3/Rgt2-Rgt1 (SRR) pathways and glycolysis in yeast resulting in *HXT2* gene repression, under fermentable and non-fermentative growth conditions. Transcription of the *HXT2* gene is repressed by the Rgt1/Ssn6-Tup1 complex in the absence of glucose and under non-fermentable growth conditions (A). Rgt1 recruits Ssn6-Tup1 in a Mth1-dependent manner to form the repressor complex. Mth1 protein prevents Rgt1 phosphorylation by PKA kinase. Snf1 kinase is activated under these conditions [33]. Snf1 kinase phosphorylates and regulates the Mig1 interaction with DNA and Ssn6-Tup1. See the text for further details. LEGEND: Fps1- glycerol transporter/exporter, Stl1 –glycerol transporter [57], Gut1- glycerol kinase converts glycerol to glycerol-3-phophate. (B) Under high-glucose conditions, the *HXT2* gene is repressed by the Snf1-Mig1 pathway. Mig1 is dephosphorylated and recruits Ssn6-Tup1 to mediate the repression. Snf1 induces the repression of *MTH1* gene expression. Glucose binding generates a signal that leads to proteasomal degradation of Mth1 *via* the Snf3/Rgt2-Rgt1 (SRR) pathway, which causes Rgt1 phosphorylation by PKA. See the text for further details. LEGEND: Yck1, 2 casein kinases, Std1- SCF^Grr1^ complex, Hxk2- glucose hexokinase, Reg1-Glc7- phosphatase complex. The diagrams are presented in SBGN format using CellDesigner^™^ 4.4 software.

Snf3, and Rgt2 the components of the glucose induction signaling pathway, are the glucose protein sensors embedded in the cell membrane [11], [12]. Binding of glucose to sensory proteins outside the cell activates the downstream signaling cascade within the cell that leads to the activation of *HXT* genes expression encoding glucose transporters of different affinity to glucose (Fig 1).

The sensors also provide information about the concentration of available extracellular glucose. Snf3 senses low availability of glucose, which leads to expression of *HXT* genes with high/intermediate affinity for glucose (*HXT6*, *HXT7*, *HXT2* and *HXT4*). Rgt2 initiates low-affinity signaling and glucose uptake system *via* the *HXT1* and *HXT3* transporters when glucose is abundant [13], [14]. *HXT2* gene expression, which responds by full induction at low glucose concentration, shows an opposite behavior in response to the glucose to *HXT1*, which is fully activated at high glucose concentration in the medium [15], [16]. *HXT*6 and *HXT7* are the only genes that encode glucose transporters, the expression of which increases not only under glucose-limited conditions but also in the presence of a non-fermentable carbon source, such as ethanol or glycerol [15], [17].

The regulation of *HXT* expression has been reported to be extremely sensitive and responsive to subtle changes in the levels of the components of signaling cascade [18]. Mth1 and Std1 corepressor proteins are the only reported links in the signaling cascade between the transmembrane glucose sensor proteins Snf3/Rgt2 and the transcription factors that physically bind DNA to regulate the expression of genes, whose products are responsible for glucose uptake and metabolism [19], [20]. Mth1 and Std1 are mainly functional in the absence of glucose. Mth1 is abundant only under conditions of no external glucose. External glucose when properly sensed, initiates Mth1 degradation *via* SCF^Grr1^ complex mediated ubiquitination and the 26S proteasome which is triggered by Mth1 phosphorylation by Yck1 and Yck2 casein kinases and other unknown factors [21], [22], [23].

The lack of *MTH1* gene has been shown to have no effect on the expression of *HXT1* encoding a low-affinity transporter but strongly increases the expression of *HXT2* in the absence of glucose, on a non-fermentable carbon source [24], [25]. Std1, which has been established as functionally redundant to Mth1, has a strong inhibitory effect on *HXT1* and *HXT3* transcription and its effect on *HXT2* and *HXT4* is negligible [24], [26].

Mth1 facilities the transcriptional repression of *HXT* genes by regulating the function of the Rgt1 transcriptional repressor by preventing its phosphorylation by PKA kinase (cAMP-PKA pathway master component) in the nucleus, in the absence of glucose [27], [19], [20], [28], [9], [29], [30]. Therefore Mth1 and Rgt1 serve as regulators which connect the Snf3/Rgt2 and cAMP-PKA pathways.

Dephosphorylated Rgt1 remains bound to DNA until Mth1, which forms a corepresor complex with Rgt1, is present [31], [32], [19]. Mth1 seems to be a “central signal receiver” in the network of three intertwining signaling pathways Snf1-Mig1, Snf3/Rgt2-Rgt1 (SRR) and cAMP-PKA pathways and the combination of Mth1 and Rgt1 corepressors is required for full *HXT2* repression [18]. Keeping the level of corepressors is crucial to maintain repression. *MTH1* gene expression is downregulated by glucose through the Snf1-Mig1 signaling pathway [20].

The Snf1-Mig1 signaling is a well-established pathway for transducing information about the intracellular environment to the glucose-repressed genes and it is intertwined with the glycolytic pathway through Snf1 kinase, which is constitutively associated with hexokinase 2 (Hxk2) protein [33], [34], [35]. Hxk2 has a dual function: in addition to phosphorylating glucose in the first catalytic reaction of the glycolytic pathway, it is an important regulator of the glucose repression signal in the nucleus. Recently, Hxk2 has been proposed as an intracellular glucose sensor that changes its conformation in response to cytoplasmic glucose levels [36].

The precise regulatory role of Hxk2 in the glucose signaling network remains obscure. The Snf1 kinase, the budding yeast orthologue of AMP-activated protein kinase (AMPK), is a key component of the glucose repression pathway and regulates only a limited set of glucose-repressed genes [32], [16], [37]. When activated in the absence of glucose, Snf1 is able to phosphorylate Mig1 and permits expression of the genes under Mig1-mediated glucose repression [38], [3], [27], [39]. Mig1 regulates the majority of glucose-responsive genes to fulfill the activities needed for ethanol metabolism, β- oxidation of fatty acids, amino acids transport and biosynthesis, meiosis and sporulation, however it is also targeted to *HXT* genes to prevent the unnecessary biosynthesis of glucose transporters, with Km for glucose, that is inconsistent with the current availability of the hexose concentration in the environment [39].

In pursuit of reprogramming the *HXT* genes expression, Mig1 and Rgt1, in association with their target promoters, recruit the Ssn6-Tup1 corepressor protein complex [40]. Activation of *HXT2* expression has been shown to be dependent on the release of Ssn6-Tup1 repression by Snf1 kinase [41]. Tup1 has been reported to function as a corepressor of the *HXT1* gene [42], [43], but has also been shown to be involved in the induction of genes expression through reorganization of the chromatin structure. In the newest model proposed by Wong and Struhl the Ssn6-Tup1complex has a dual function in gene transcription as a repressor-activator complex [44]. Tup1 has been shown to play a role in the recruitment of the acetyltransferase SAGA complex, the ATP-dependent chromatin remodeling SWI/SNF global transcription activator complex and the mediator to several promoters [45], [46], [43], [44].

### Activity of RNA Polymerase III in response to glucose

Yeast cell systems biology provides evidence that in response to glucose, a synergistic metabolic and transcriptional control occur, which is illustrated by the up-regulation of several genes involved in transcription and translation functions such as common subunits of RNA polymerases I, II and III as well as genes encoding products that are necessary for tRNA modification [47]. Thus, yeast modulate the level of protein synthesis accordingly to their metabolic requirements. The regulation of RNAP III is primarily tied to the regulation of cell growth and cell cycle [48]. Maf1, which is the only negative regulator of RNA Polymerase III, has been shown to be involved in coupling carbon metabolism to tRNA transcription [49]. Under unfavorable growth conditions, the Maf1 protein represses RNAP III activity [50], [51]. In yeast the regulation of tRNA transcription by Maf1 is essential at the transition from fermentative growth on glucose to oxidative respiratory growth. Maf1 plays an essential mediator role integrating carbon metabolism with repression of RNAP III dependent transcription and is broadly conserved in eukaryotes [52], [53], [54].

Inactivation of the gene encoding Maf1, up-regulates tRNA synthesis and inhibits yeast growth on respiratory substrates, which is correlated with a decrease in *FBP1* and *PCK1* gene transcription, the genes indispensable for utilization of non-fermentable carbon source [55]. The growth defect in *maf1-Δ* on glycerol can be suppressed by mutations that down-regulate RNAP III transcription. The *rpc128-1007* mutant carrying a single nucleotide change localized close to the nucleotide binding motif in the C-terminus of the RNAP III catalytic subunit C128, is one of the suppressors, that restores the ability of *maf1-Δ* deficient cells ability to grow in medium with glycerol as a sole carbon source [56].

Here we study the unusual relationship between RNA Polymerase III activity and *HXT* genes expression, which are protein-coding genes dependent on RNA Polymerase II transcription.

We used two *S*. *cerevisiae* mutants, *maf1-Δ* and *rpc128-1007* with, respectively, up- and down-regulated RNAP III activity [49] to examine the expression of the high-affinity glucose transporter gene *HXT2*. In this study, we report that altered RNAP III activity in the aforementioned mutants *maf1-Δ* and *rpc128-1007* affect the levels of *HXT* mRNA encoding high-glucose affinity transporters, both on high glucose concentration and on non-fermentable carbon source.

Based on the results obtained during this study on glucose signaling *via* Snf1-Mig1, and Snf3/Rgt2-Rgt1 (SRR) systems, we discuss and provide evidence of an exceptional case of *HXT2* expression to the generalized causality model by Zaman asserting that the cellular perception of nutrient availability establishes the transcriptional pattern in yeast [16].

## Materials and methods

### Yeast strains, plasmids and media

The following yeast strains were used in this study: wild-type MB159-4D [58] with unchanged RNAP III activity, the MB159-4D *maf1-Δ*::*URA3 MAF1*-deficient mutant with elevated RNAP III activity and the MJ15-9C mutant with a single point mutation in the C128 RNAP III subunit with reduced enzyme activity (Table 1). To delete *MAF1*, an *URA3* cassette surrounded on each side by *MAF1*-flanking regions was extracted from an agarose gel after plasmid I-0306 digestion with *HindIII*, *EcoRI* and *PvuI*. The digestion product of 2200 bp was transformed into the MB159-4D background using the LiAc/SS carrier DNA/PEG method [59]. The replacement of *MAF1* with the *URA3* cassette was confirmed by PCR. Strain BY4741 *reg1-Δ*::*kanMX4* was obtained from Euroscarf. The Y0753 *rsp5-Δ*::*HIS3* [SPT23^1-686^:URA3] transformant was obtained from the Institute of Biochemistry and Biophysics, PAS, Warsaw. The strains were used for RNA preparation, real-time PCR quantification and glucose uptake analysis.

**Table 1:** Yeast strains used in the study.

Strain	Genotype	Reference/ Source
MB159-4D	*MATa SUP11 ura3 leu2-3*, *112 ade 2–1 lys 2–1 trp*	[58]
MA159-4D *maf1-Δ*	*MATa SUP11 ura3 leu2-3*, *112 ade 2–1 lys 2–1 trp maf1-Δ*::*URA3*	This study
MJ15-9C	*MATa rpc128-1007 SUP11 ura3 leu2-3*, *112 ade 2–1 lys 2–1 trp*	[49]
MA159-4D Mig1-3HA	*MATa SUP11 ura3 leu2-3*, *112 ade 2–1 lys 2–1 trp Mig1-3HA-kanMX6*	This study
MA15-9C Mig1-3HA	*MATa rpc128-1007 SUP11 ura3 leu2-3*, *112 ade 2–1 lys 2–1 trp Mig1-3HA-kanMX6*	This study
MA159-4D Tup1-3HA	*MATa SUP11 ura3 leu2-3*, *112 ade 2–1 lys 2–1 trp Tup1-3HA-kanMX6*	This study
MA15-9C Tup1-3HA	*MATa rpc128-1007 SUP11 ura3 leu2-3*, *112 ade 2–1 lys 2–1 trp Tup1-3HA-kanMX6*	This study
MA159-4D Rgt1-3HA	*MATa SUP11 ura3 leu2-3*, *112 ade 2–1 lys 2–1 trp Rgt1-3HA-kanMX6*	This study
MA15-9C Rgt1-3HA	*MATa rpc128-1007 SUP11 ura3 leu2-3*, *112 ade 2–1 lys 2–1 trp Rgt1-3HA-kanMX6*	This study
RS159-4D Mth1-3HA	*MATa SUP11 ura3 leu2-3*, *112 ade 2–1 lys 2–1 trp Mth1-3HA-kanMX6*	This study
RS15-9C Mth1-3HA	*MATa rpc128-1007 SUP11 ura3 leu2-3*, *112 ade 2–1 lys 2–1 trp Mth1-3HA-kanMX6*	This study
Y0753 *rsp5-Δ*	*MATa*, *his3Δ200 leu2-3*, *2–112*, *lys2-901*, *trp1-1*, *ura3-52 rsp5-Δ*::*HIS3 [SPT23*^*1-686*^:*URA3]*	[54]
BY4741 *reg1-Δ*	*MATa his3Δ1 leu2Δ0 met15Δ0 ura3Δ0 reg1-Δ*::*kanMX4*	Euroscarf
BY4741 *fab1-Δ*	*MATa his3Δ1 leu2Δ0 met15Δ0 ura3Δ0 fab1-Δ*::*kanMX4*	Euroscarf
BY4741 *vac14-Δ*	*MATa his3Δ1 leu2Δ0 met15Δ0 ura3Δ0 vac14-Δ*::*kanMX4*	Euroscarf

The Mig1-3HA, Rgt1-3HA, Tup1-3HA and Mth1-3HA chromosomally encoded yeast derivatives were obtained by PCR amplification of the KanMX6 cassette from pFA6a-3HA-kanMX6 plasmid DNA. The primers used for amplification were designed to create C-terminal 3HA epitope-tagged proteins according to Longtine [60]. MB159-4D and the MJ15-9C were transformed with PCR products and after verification of positive clones by control PCR were used for Western blotting and chromatin immunoprecipitation (ChIP). The primers used to generate 3HA tagging and plasmids are listed in Supporting information S1 and S2 Tables.

All yeast strains were prepared according to Sherman [61]. Rich yeast YP medium (1% peptone, 1% yeast extract) was supplemented with 2% glucose (YPD) or 2% glycerol (YPGly) as a carbon source. For transformation purposes the transformants were selected on the YPD medium supplemented with G418 sulfate (geneticin) or SC minimal medium (0.67% yeast nitrogen base, 0.77 g/L appropriate amino acids CSM) was supplemented with 2% glucose medium. Yeast cells transformation mixtures were cultivated on plates containing SC medium lacking uracil and YPD supplemented with geneticin at 30°C for 3 days.

For other experiments, overnight yeast cultures were diluted to A_600_ ≈ 0.1 and grown to logarithmic phase (A_600_ ≈ 1.0) at 30°C with agitation at 250 rpm in YPD or YPGly. When *maf1-Δ* grown in YPGly medium and when reached A_600_ ≈ 1.0 it was shifted from 30°C to 37°C for 2 h.

### Preparation of RNA and real-time PCR quantification

The MB159-4D reference strain and the *rpc128-1007*, *maf1-Δ* and *rsp5-Δ* [SPT23^1-686^:URA3] mutant strains were cultured in liquid medium in triplicate as independent biological repeats. The isogenic wild-type strain and *maf1-Δ* and were grown in YPGly medium with 2% glycerol to log phase (A_600_ ≈ 1.0) and then shifted to the restrictive temperature (37°C) for 2 h, allowing *maf1-Δ* to accumulate tRNA. The cultures (20 ml) were harvested by centrifugation and rapidly frozen in liquid nitrogen. RNA isolation was performed according to Schmitt [62] protocol with increased phenol: chloroform: isoamyl alcohol volumes (500 μl of the mixture). Total RNA was isolated by heating and freezing the cells in the presence of sodium dodecyl sulfate (SDS) and phenol. Cells were resuspended in 50 mM Na acetate pH = 5.3 10 mM EDTA. The incubation time with phenol at 65°C was extended to 5 min. Additionally, glass beads (425–600 μm; Sigma Aldrich) were used for cell disintegration. The samples were homogenized using the MiniBeadbeater 24 (Biospec products) 3 times using 3000 hits per minute with a duration of 20 s each. Total RNA was quantified spectroscopically using an automated analyzer Synergy H4 (BioTek), and its integrity was assessed by gel electrophoresis in 1x NBC (0.05 M boric acid, 0.1 mM Na citrate, 5 mM NaOH with formaldehyde). One microgram of isolated RNA was treated with DNAse I (ThermoScientific) according to the manufacturer’s suggested protocol. The reverse transcription reaction was primed using random hexamer oligonucleotides. Real-Time PCR reaction was performed in 384-well plates using the LightCycler 480 instrument (Roche Molecular Biochemicals). The reaction mixture (10 μl volume) was prepared with the following components: 5 μl of 2x Kapa SYBR Fast qPCR MasterMix, 3 μl of 1 mM forward and reverse gene-specific primers mix and 2 μl of cDNA. The amplification reaction consisted of 3 min of preincubation in 95°C and 45 cycles of amplification at 95°C for 10 s, 60°C for 15 s and 72°C for 15 s. At the end of the analysis, the amplification specificity was controlled by subjecting the products to a melting curve analysis in which the temperature was increased from 65°C to 97°C at 2.2°C/s. The threshold (Ct) values were obtained using the automated setting of the LightCycler software release 1.5.0 (Roche Molecular Biochemicals). The samples were normalized to two reference genes—U2 spliceosomal RNA (*U2*) and small cytosolic RNA (*SCR1*) by using the Vandesompele method [63]. To identify the best reference genes among *U2*, *SCR1*, *PGK*, *QCR9*, the NormFinder macro was applied using the algorithm proposed by Andersen [64]. All of the reaction sets were conducted at least in triplicate and each set included a no template control. The relative expression levels of the chosen genes and standard deviation were calculated using the Gene Expression Analysis for iCycler iQ^®^ Real-Time PCR Detection System macro version 1.10 (Bio-Rad, 2004) according to Livak method [65]. The statistical significance was computed using one-way completely randomized ANOVA. The oligonucleotides used for real-time PCR are listed in Supporting information S3 Table.

### Western blotting and immunoprecipitation

Rgt1-3HA and Tup1-3HA-expressing strains were grown to A_600_ ≈ 1.0, centrifuged, rapidly frozen in liquid nitrogen and stored at -20°C. For Mig1-3HA analysis, yeast cultures at A_600_ ≈ 1.0 were quenched with 100% trichloroacetic acid solution (TCA) at a ratio of 1:5 before centrifugation [66]. To extract proteins, yeast pellets were incubated in 2 M NaOH supplemented with 7% β- mercaptoethanol for 2 min, followed by addition of 50% TCA, vigorously mixing and centrifugation. Cell pellets were washed twice with 1 M Tris-HCl pH = 7.5 buffer. Protein extracts were suspended in 2x sample buffer (2x SB 0.1 M Trisma base pH = 8.8, 4% SDS, 20% glycerol, 7% β- mercaptoethanol, 0.008% bromophenol blue), boiled at 95°C for 5 min, separated by 10% SDS-PAGE, electrotransferred to a nitrocellulose membrane and hybridized with mouse monoclonal anti-HA antibody (Covance, MMS-101P-1000; AB291259) diluted 1:2000 for 1 h. The nitrocellulose membrane was then incubated with secondary polyclonal goat anti-mouse antibodies conjugated to horseradish peroxidase (Dako, P0447; AB 2617137) diluted 1:2000 for 1 h. A 1:4000 dilution of mouse anti-Vma2 (LifeTechnologies, A6427; AB 2536202) was used for quantification purposes. Membranes were developed using the Clarity Western ECL chemiluminescence detection kit (Bio-Rad, 170–5060). To visualize Mth1, total protein extracts were obtained from 100 ml cultures expressing Mth1-3HA. The cells were suspended in a lysis buffer (50 mM Tris-HCl pH = 7.5; 1% Triton X-100; 250 mM NaCl with Protease Inhibitor Cocktail Tablets, Roche) and disrupted by vortexing with glass beads (425–600 μm; Sigma Aldrich) using 7 bursts of 1 min each at a settings of 3000 rpm/min. The samples were cooled on ice for at least 2 min between bursts. Protein extracts were immunoprecipitated with magnetic beads (Dynabeads Pan Mouse IgG, Invitrogen by Life Technologies) conjugated to mouse monoclonal anti-HA antibodies (Covance, MMS-101P-1000; AB 291259) at a dilution of 1:80 dilution for 18 h at 4°C. The beads were washed twice with lysis buffer, suspended in 2x SB and boiled for 10 min. The membrane was blocked with 5% milk and incubated with primary monoclonal rabbit anti-HA antibodies (Abcam, ab184643) diluted 1:4000 for 1 h, followed by incubation with secondary AP-conjugated polyclonal goat anti-rabbit antibodies (Dako, D0487; AB 2617144) diluted 1:2000 for 1 h. The BCIP/NBT Liquid Substrate System (Sigma, B1911) was used for visualization purposes. The protein bands intensity was measured using Syngene—GeneTools version 4.3.5 software.

### Chromatin immunoprecipitation (ChIP-qPCR)

Overnight yeast cultures were used to inoculate fresh YPD and YPGly medium to A_600_ *≈* 0.2. The cultures were grown to A_600_ *≈* 1.0. and fixed with formaldehyde at a final concentration of 1% for 20 min at room temperature. The reaction was stopped with glycine, which was added at a final concentration of 125 mM and incubated for 5 min to quench the cross-linking reaction. The cells were centrifuged, wash twice with 1x phosphate-buffered saline pH = 7.5 (PBS) frozen in liquid nitrogen and stored at -80°C. Yeast chromatin isolation, sonication, magnetic beads preparation, immunoprecipitation, elution and decrosslinking were performed according to Ren *et al*. with modifications [67]. The cells were disrupted with glass beads (425–600 μm; Sigma Aldrich) in FA/SDS buffer (50 mM HEPES; 1 mM EDTA pH = 8.0; 1% Triton X-100; 150 mM NaCl: 0.1% Na deoxycholate; 0.1% SDS with Protease Inhibitor Coctail Tablets, Roche) by homogenization with using a Mini-Beadbeater 24 (Biospec products) with 15 bursts of 1 min each at 3000 rpm/min at 4°C. Between bursts, the samples were cooled on ice for at least 1 min. Each tube was pierced at the bottom and the cell lysate was collected by centrifugation in a fresh tube. The lysate was sonicated 6 times for 30 s each (Bandelin Sonoplus Ultrasonic Homogenizer HD 2070, MS 72 microtip) at 20% power to obtain DNA fragments ranging in size from 0.5–1.5 kb as determined by gel electrophoresis. The genomic DNA fragments were immunoprecipitated with mouse monoclonal anti-HA antibodies (Covance, MMS-101P-1000; AB 291259) conjugated to magnetic beads (Dynabeads Pan Mouse IgG, Invitrogen by Life Technologies). IP and 1:100 diluted INPUT samples were analyzed by quantitative PCR with the SYBER GREEN system as described in real-time PCR quantification section. ChIP-qPCR calculations were conducted as described by Lin *et al*. [68]. The oligonucleotide sequences of the primers used in ChIP assay are available upon request.

### Glucose transport measurement by 2-NBDG uptake assay

Cells were grown as indicated in section 2.1 in YPD containing 2% glucose. Cells were harvested, washed twice with SC minimal medium supplemented with amino acids without a carbon source, resuspended in the aforementioned medium and incubated for 10 min at 30°C with agitation.

High-affinity glucose transport was measured using 2-[*N*-(7-nitrobenz-2-oxa-1,3-diazol-4-yl)amino]-2-deoxy-D-glucose (2-NBDG, Invitrogen) at a final concentration of 1 mM [69]. The uptake assay was performed over time. Samples were collected directly after 2-NDBG addition at time = 0, after 0.5 min, 2.5 min, 5 min, 10 min, 15 min and 20 min. At each time point cell suspensions exposed to 2-NBDG, were immediately transferred to fresh tubes containing 10% formalin, fixed, washed and suspended in 1x phosphate-buffered saline pH = 7.5. Fluorescence was determined using and automated analyzer for spectroscopic measurements Synergy H4 (BioTek) at excitation 490 nm and emission 516 nm. The mean fluorescence intensity (MFU) of intracellular 2-NBDG was calculated and normalized by subtracting the background autofluorescence signal measured for the cells suspensions without 2-NDBG. Four independent experiments were performed.

### Enzymatic assays

#### Invertase activity assay

Yeast cultures grown in YP medium supplemented with either 2% glucose or 2% sucrose medium, were harvested at A_600_ ≈ 1.0. Cell suspensions were prepared according to the protocol described by Silveira *et al*. [70]. Invertase activity was measured using the commercially available Invertase Activity Colometric detection Kit (BioVision Incorporated, Cat. K674-100). Measurements were performed according to the manufacturer’s protocol with modifications. Cells were disrupted using glass beads (425–600 μm; Sigma Aldrich) at 8 bursts of 10 s each using a Mini-Beadbeater 24 (Biospec products). In between the bursts, samples were cooled on ice for 1 min. Protein concentrations were measured using Bradford Reagent (Sigma, B6916) [71]. Four independent biological replicates were assessed. The invertase activity is expressed as μmol of substrate converted per min per mg of extracted protein (μmol of glucose·min^-1^·mg^-1^ protein).

#### Determination of glucose concentration in the medium

To measure glucose depletion in a yeast culture over time, aliquots were collected at regular intervals from cultures starting from *A*_*600*_ = 0.2 and grown until *A*_*600*_ = 1.4. The enzymatic reactions were prepared and rates measured in NADH-liked assays according to the manufacturer’s protocol supplied with the Glucose (HK) Assay Kit (Sigma, GAHK-20). The change in NADH concentration over time in each reaction was monitored by measuring the absorbance at 340 nm using an automated analyzer for spectroscopic measurements Synergy H4 (BioTek). Three independent biological replicates were assessed. The glucose concentration is expressed as mg of glucose in 1 ml of culture medium.

## Results

### The transcription of *HXT* genes encoding high-affinity transporters is affected in mutants with altered RNAP III transcription

Maf1-deficient cells (Table 1) seem to efficiently utilize glucose, but they are not able to grow on a non-fermentable carbon source at elevated temperatures. The defect in *maf1-Δ* growth is suppressed by the second-site suppressor *rpc128-1007* (Table 1), which carries a point mutation in the C-terminal portion of the second largest RNAP III yeast subunit, C128 [49]. The *rpc128-1007* strain was found to efficiently utilize glycerol as a carbon source, but it poorly grows on 2% rich glucose medium (at high glucose concentration) [49].

We initiated our study to investigate the regulation of high affinity glucose transporters expression in these strains, showing different RNAP III activities, guided by previous transcriptomics data, which suggested that the expression profiles of *HXT6*, *HXT7*, and *HXT2* genes are altered in the *maf1*-*Δ* mutant grown in medium containing glycerol [55].

*maf1-Δ* was grown on glucose or glycerol-supplemented medium at 30°C and shifted to 37°C for 2 h, thus under growth conditions in which the mutant exhibits impaired gluconeogenesis [49], [55]. Initially, we aimed to investigate the expression of the *HXT6* and *HXT7* genes separately, however due to nearly identical ORF sequences (99.7% identity) [72] this attempt was withdrawn. Therefore, we present a summary of the results for the transcription of the both genes. Real-time PCR was performed on cDNA using two internal controls, *SCR1* and *U2*.

**Fig 2:**
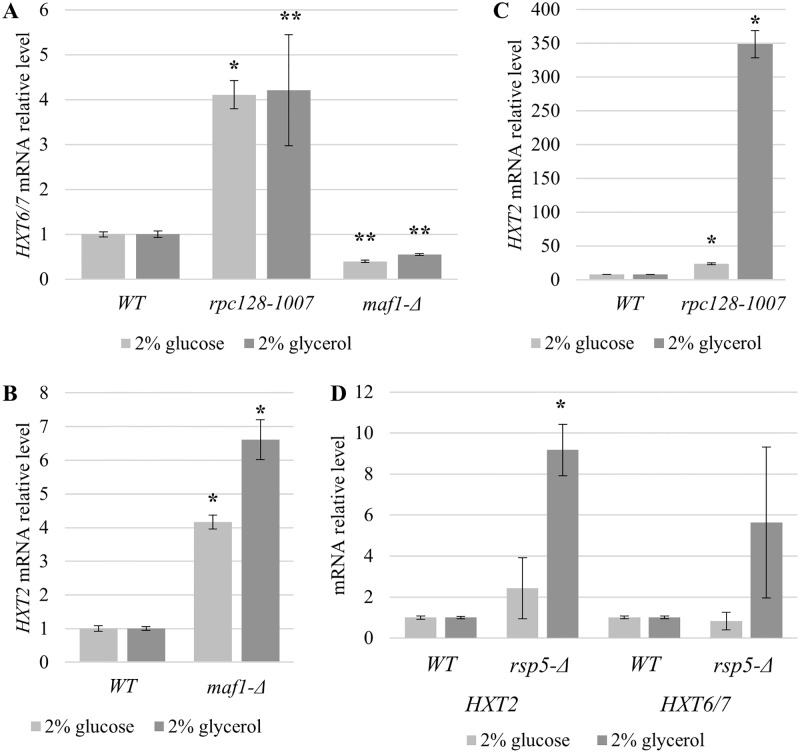
mRNA levels of *HXT6*/7 and *HXT2* in strains with different RNAP III. *HXT6*/7 in *maf1-Δ* and *rpc128-1007* (A), *HXT2* in *maf1-Δ* (B), *rpc128-1007* (C) and *rsp5-Δ* [SPT23 ^1–686^:URA3] (D) yeast strains. Strains were cultured in rich medium (YP) supplemented with either 2% glucose or 2% glycerol. Additionally, *maf1-Δ* cells grown on glycerol were shifted to 37°C for 2 h. RNA was isolated when the culture reached A_600_ ≈ 1 and reversed-transcribed to cDNA. Isolated RNAs were examined by SYBR GREEN-based real-time PCR. The samples were normalized to two reference genes: *U2* spliceosomal RNA (*U2*) and small cytosolic RNA (*SCR1*). The bars represent ratios between the levels of respective mRNAs in the mutants and the control isogenic wild-type strain. The expression level in the WT strain (MB159-4D) was set as 1.0. The means ± standard deviations of the relative expression levels are shown. Asterisks (*) indicate p-values ≤ 0.05 determined by one-way ANOVA. Double asterisk (**) indicate p-values ≤ 0.15.

As shown in Fig 2, the *HXT6/7* transcript levels in *maf1-Δ*, grown on either glucose or glycerol-supplemented medium, were lower than those obtained for the parental strain (0.40-fold lower and 0.55-fold lower, respectively) (Fig 2A). In contrast, we observed an increase in *HXT6/7* levels in *rpc128-1007* under the same conditions by 4.22-fold on glycerol and 4.11-fold on glucose-rich medium. Thus, the transcription of *HXT6/7* seems to be anti-correlated with RNAP III activity.

This observation prompted us to examine the transcription of *HXT2*, another gene that is transcriptionally regulated *via* glucose repression mechanism [73], [8], [74].

In this case, real-time-PCR revealed that *HXT2* transcript levels were elevated by 6.61-fold when *maf1-Δ* was grown on glycerol. Unexpectedly, the *HXT2* gene’s expression was elevated by 4.16-fold compared with the control strain when grown in the presence of 2% glucose (Fig 2B).

Next, we verified how *rpc128-1007*, which causes a decrease in RNAP III activity, affects the level of *HXT2* under high-glucose conditions and on a non-fermentable carbon source. In the *rpc128-1007* mutant, the *HXT2* transcript levels were 23-fold higher in the presence of 2% glucose and even more elevated, by 340-fold, when glycerol was the only available carbon source in the medium (Fig 2C). These results suggest that the defect in RNAP III transcription caused by the genetic mutation in C128 RNAP III subunit fully de-represses *HXT2* regardless of the carbon source. We excluded the possibility that glucose was exhausted during yeast cultivation by measuring external glucose concentrations in the medium (S1 Fig). The mutated strain becomes insensitive to the external glucose availability, raising the question whether this effect is caused by the specific mutation in RNAP III or it is rather related to the growth features of the mutant. The growth rate of *rpc128-1007* is low, particularly in the presence of glucose. The relation of growth rate to induction of glucose repressed genes was suggested by Zaman and coworkers in 2009 [16].

To address this issue, we examined *HXT2* gene expression in another strain with a poor growth rate comparable to *rpc128-1007*, which was an *rsp5-Δ* mutant (Table 1).

Rsp5 protein is an ubiquitin ligase that is engaged in cellular processes such as regulating the trafficking of nutrient permeases and transporters in response to environmental changes [75], [76], [77]. It is not related to RNAP III activity. Deletion of the *RSP5* gene affects the actin cytoskeleton organization and endocytosis of Hxt1 and Hxt3 transporters [78]. Rsp5 is also implicated in the activation of the plasma membrane H^+^-ATPase Pma1 by glucose [79], but the mechanism underlying this regulation is not known. The growth rate of the *rsp5-Δ* [SPT23^1-686^:URA3] strain, used in this study is comparable to *rpc128-1007* (S2 Fig). Overexpression of a truncated clone of Spt23 allows *rsp5-Δ* to growth without oleic acid supplementation, because the deletion of *RSP5* affects fatty acids biosynthesis by reducing *OLE*1 expression.

We observed an increase in *HXT2* mRNA levels in *rsp5-Δ* but the fold change in expression was several times lower than that measured in *rpc128-1007* both on glucose and glycerol (Fig 2D). Altogether, we concluded that high *HXT2* transcript levels result from downregulation of RNAP III activity, rather than by a general growth defect.

This observation primarily suggested that changes in RNAP III activity lead to aberrant glucose sensing or signaling, and thus we focused on elucidating the cause of the phenomenon using the *rpc128-1007* mutant and the *HXT2* gene as a model. We verified repressor/activators proteins levels in the mutant cells, their occupancy on *HXT2* gene and examined posttranslational status of the regulatory player according to available literature data accumulated since 1990s.

### Glucose uptake is sustained in strains with different RNAP III activities

Since the *HXT2* and *HXT6/7* genes are transcribed more efficiently in *rpc128-1007* and there is a clear difference in carbon source preference for the *maf1-Δ* mutant with high RNAP III activity, we further explored a link between the glucose transport efficiency and RNAP III transcription.

We performed a non-radioactive glucose uptake assay using fluorescent glucose analog 2-NBD to measure time-dependent glucose uptake in RNAP III mutants. We focused the analysis on high-affinity glucose transporters guided by the real-time PCR data for the expression profiles of the *HXT6*, *HXT7* and *HXT2* genes.

The ability of the strains to take up glucose was monitored after sudden relief from glucose limitation by addition of 2 mM 2-NBD glucose analog.

**Fig 3:**
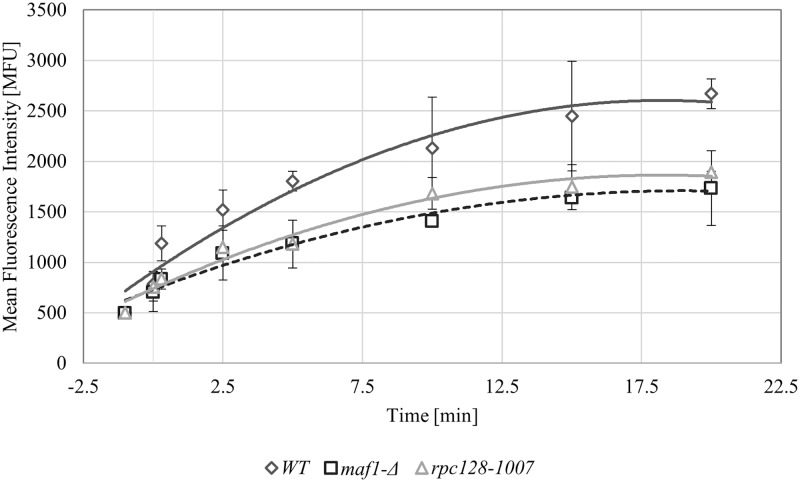
Glucose flux is present in all tested strains. High-affinity glucose transport was measured in the WT (MB159-4D), *rpc128-1007* and *maf1*-Δ strains. The assay was performed using fluorescently labeled glucose 2-NDBG. The cells were grown to exponential phase of *A*_*600*_ ≈ 1.0 in 2% glucose-rich medium (YPD), transferred to SC medium supplemented with amino acids without a carbon source and incubated for 10 min at 30°C. The uptake of 2-NDBG (1 mM concentration) was performed over time. As a control, cell suspensions without fluorescently labeled glucose were assessed. The results are expressed as the mean fluorescence intensity (MFU) of four independent biological replicates with standard deviations.

The wild-type strain showed the most efficient glucose uptake among the three tested strains. As shown in Fig 3, the second most efficient strain was the *rpc128-1007* strain, which has reduced RNAP III activity. The *maf1-Δ* mutant with the highest RNAP III activity was the least efficient for uptake in the presence of low external glucose concentrations, which involves inducing the expression of the high-affinity glucose transporter.

Despite differences in glucose uptake by the tested strains, the influx of glucose into the cells seems to be sustained in all cases.

### Mig1 binds to the *HXT2* promoter accordingly to the factual, high glucose availability, but does not repress the gene in cells with low RNAP III activity

A bottom-up approach was undertaken to elucidate the cause of the glucose repression relief in the *rpc128-1007* mutant.

**Fig 4:**
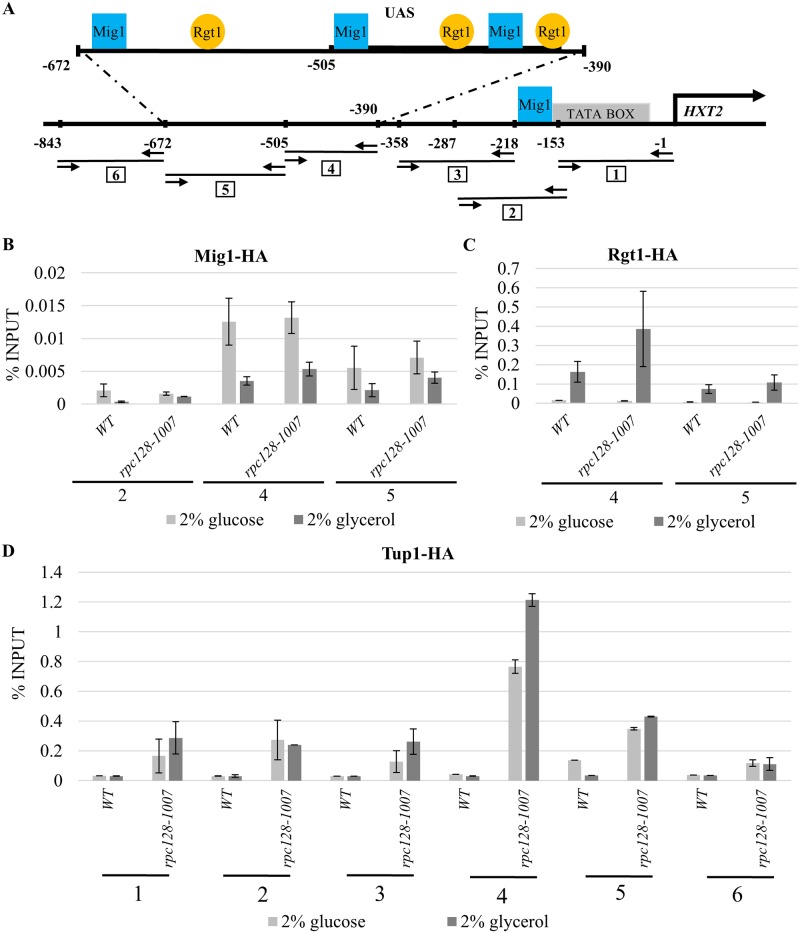
Tup1 occupancy is significantly increased on the transcriptionally active *HXT2* promoter in *rpc128-1007*. (A) Schematic representation of the *HXT2* promoter region. The Rgt1 binding site is indicated by a circle. The Mig1 binding site is denoted by a square. The horizontal lines represent cDNA fragments amplified by real-time PCR in the ChIP assay (numbered in squares). Arrows facing each other correspond to primer pairs hybridization. Coordinates represent the position of the aforementioned elements in the region with respect to the translation start site. The black arrow above *HXT2* indicates the translation start site. The WT (MB159-4D) and *rpc128-1007* strain expressing HA-tagged Mig1 (B), HA-tagged Rgt1 (C) and HA-tagged Tup1 (D) were grown in 2% glucose or 2% glycerol-rich medium. Crosslinked chromatin was immunoprecipitated with antibodies against the HA epitope, followed by real-time PCR. The signals are presented as the percent of the INPUT signal from three separate experiments with standard deviations. Numbers from 1 to 6 correspond to real-time PCR amplification products in the *HXT2* promoter region on the A panel. (B) Mig1 shows the same degree of occupancy on the *HXT2* promoter in the wild-type strain and mutant strain with low RNAP III activity at high glucose conditions. (C) Rgt1 had a higher occupancy on the *HXT2* promoter in the *rpc128-1007* strain with low RNAP III activity. (D) Tup1 shows enhances association with the *HXT2* promoter in *rpc128-1007* despite the carbon source.

**Fig 5:**
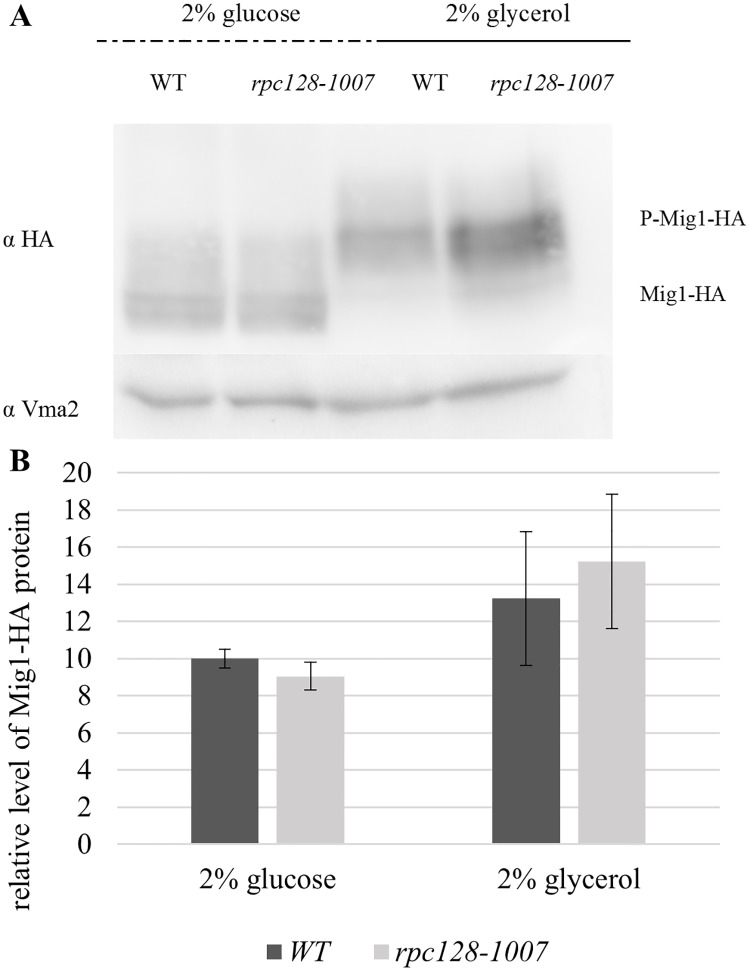
Mig1 cellular concentration and phosphorylation pattern under different growth conditions. The WT strain (MB159-4D) and *rpc128-1007* mutant strain expressing HA-tagged Mig1 proteins were grown to A_600_ ≈ 1.0 in 2% glucose or 2% glycerol-rich medium. Total cell protein extracts were subjected to SDS-PAGE and examined by Western blotting using anti-HA antibodies (A) P-Mig1-HA: phosphorylated Mig1-HA. The quantified relative level of Mig1–HA (B) protein compared to yeast Vma2 protein was calculated for at least three independent experiments conducted in triplicates with standard deviations. The molecular weight (MW) of Mig1-HA was ~ 64.8 kDa the MW of Vma2 was ~ 57.7 kDa.

The *HXT2* promoter is a carbon source-dependent promoter and in yeast [80] contains four binding sites for Mig1 repressor, the transcription factor which activity is regulated in Snf1 kinase dependent phosphorylation reaction, coordinates the expression of the majority of glucose repressed genes [38], [3], [27], [39] (Fig 4A). Constitutive hyperactivation of Snf1 kinase in *reg1-Δ* mutant results in Mig1 constitutive phosphorylation that prevents the down-regulation of *HXT2* on high glucose [73]. To test the possibility, that Mig1 is not activated in the *rpc128-1007* mutant on glucose we aimed to establish the effect of the *rpc128-1007* mutation on Mig1 occupancy of the *HXT2* promoter by chromatin immunoprecipitation (ChIP). To quantify Mig1 occupancy on the DNA, we constructed a strain chromosomally expressing 3HA epitope-tagged Mig1 (Table 1). Mig1 abundance estimated by Western blotting revealed, more Mig1 protein in cells grown on glycerol medium in comparison to favorable growth conditions. We noticed no differences in protein concentration when we compared Mig1-3HA expressed in *rpc128-1007* to its parental strain (Fig 5A and 5B). The molecular mass of Mig1 clearly suggested that the protein was in a non-phosphorylated state in the presence of 2% glucose (repression condition) in both strains. Moreover, in both strains, Mig1 efficiently underwent phosphorylation when switched to a non-fermentable carbon source (Fig 5A). These results suggest that the mechanism of Snf1 deactivation in the presence of glucose and activation in the presence of glycerol is the same in the *rpc128-1007* mutant strain and the isogenic wild-type control.

We presumed that Mig1 repressor would be bound to *HXT2* DNA on 2% glucose. To confirm this hypothesis, we performed an *in vivo* ChIP analysis of the strain grown under derepressing and repressing growth conditions in order to measure Mig1 occupancy on the DNA. Extracts of cells encoding Mig1-3HA were immunoprecipitated with anti-HA antibodies, and cDNA was subjected to analysis by real-time PCR analysis. The *HXT2* regulatory region was analyzed by the amplifications of three separated sequence fragments (marked 2, 4, 5) as indicated in the Fig 4A, which cover all the binding motifs recognized by Mig1. In all studied cases, we only observed differences in Mig1 occupancy on *HXT2* according to the carbon source. This effect occurred regardless of the presence of the *rpc128-1007* mutation. Consequently, we observed higher Mig1 occupancy at the *HXT2* gene on glucose compared with glycerol. As shown in Fig 4B, we found approximately 3-fold less Mig1 bound to the promoter under conditions of a non-fermentable carbon source, which is consistent with published data regarding Mig1 binding to promoters of glucose-repressed genes [81]. Taking together, observation of the phosphorylation status of Mig1 and its DNA binding activity in *rpc128-1007* compared with the control strain, indicated that glucose signal forwarded *via* Snf1 downstream to Mig1 could not account for the significant increase in *HXT2* expression observed on both carbon sources in cells carrying a mutated C128 RNAP III subunit. Although Mig1 bound to the *HXT2* gene under high-glucose conditions to the same extent as in the isogenic wild-type strain, this binding was not sufficient to block gene expression in the *rpc128-1007* mutant (Fig 2C).

### Snf1 dependent glucose repression is not perturbed in cells with different RNAP III activity

Since *rpc128-1007* grows poorly at high concentration of extracellular glucose (2%) we hypothesized that there was a lower glycolysis efficiency in the strain compared with the wild-type and *maf1-Δ* which in contrast displays growth inhibition on a non-fermentable carbon source but seems to metabolize glucose very efficiently when glucose is present at high concentrations [49], [82]. We verified energy status in the strains by measuring invertase activity.

Invertase activity (indispensable for sucrose utilization) is a well-established indicator of the status of glucose repression in yeast cells. It has been indicated that a regulatory mechanism that controls *SUC2* gene expression is similar to those described for the *HXT2* and *HXT4* genes [73]. High invertase activity correlates with reduced glucose transport capacity to the favor of increased transport of the alternative carbon source such as sucrose. New evidence provided by Gancedo *et al*. 2015 [83] suggests that both the Snf1-Mig1 and the Snf3/Rgt2-Rgt1 (SRR) signaling pathway are equally required to regulate the expression of the *SUC2* gene [84], [80]. A double mutant, *snf3-Δ rgt2-Δ* grows poorly on glucose and is defective in glucose repression of *SUC2* [85].

Given the results of *in vivo* ChIP assay using Mig1-3HA, it was tempting to investigate whether there is the glucose repression established properly in strains with different RNAP III activity under high glucose conditions.

The activity assay was performed under derepressing and repressing conditions with cell extracts from cultures grown on glucose and on sucrose as an alternative carbon source.

**Fig 6:**
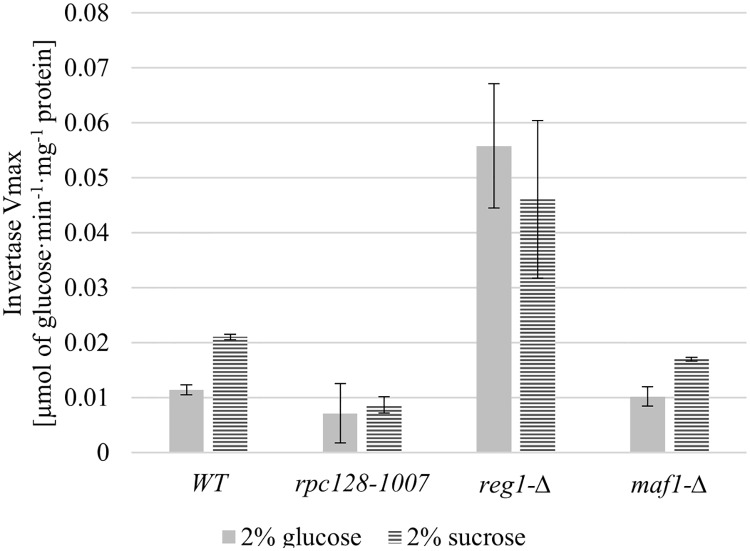
RNAP III changed activity does not affect glucose repression. The WT strain (MB159-4D) and the *rpc128-1007*, *reg1-Δ* and *maf1-Δ* mutant strains were grown under repressing (2% glucose) and activating (2% sucrose) conditions. The experiment was performed in cell-free extracts isolated from the aforementioned strains. Invertase activity is expressed in μmol of glucose·min^-1^·mg^-1^ protein. Data are expressed as the mean obtained from at least three independent experiments measured in triplicate. The ± standard deviations are shown.

As shown in Fig 6, the activity of invertase decreases in all strains, excluding *reg1-Δ* in the presence of glucose (Table 1). *reg1-Δ* was used herein as an internal positive control because, in *REG1*-deficient cells, *SUC2* is no longer regulated by glucose and is fully de-repressed due to the constitutively activated Snf1 kinase. Low invertase activity detected in *rpc128-1007* on high glucose is strong evidence that increased *HXT2* expression under the aforementioned conditions is not the result of a perturbation of the glucose repression mechanism *via* the Snf1 pathway.

### Rgt1 occupancy on *HXT2* gene is higher in the compromised RNAP III cells on non-fermentable carbon source

Since Snf1-Mig1 signaling appears to regulate a wide spectrum of genes, the Rgt1 network regulates a narrow group of genes consisting mostly of hexose transporter genes [39]. Three binding sites have been reported for the Rgt1 transcriptional repressors identified on the *HXT2* promoter [27], [20], [86] (Fig 5). Rgt1 is the transcriptional factor that receives information regarding the external glucose concentration *via* Snf3/Rgt2-Rgt1 (SRR) signaling pathway and the cAMP-PKA pathway to regulate *HXT* expression. Therefore, we examined the interaction of Rgt1 with the *HXT2* promoter by ChIP assay *in vivo*, and the phosphorylation status and abundance were determined using a set of strains expressing the Rgt1-3HA protein fusion (Table 1).

**Fig 7:**
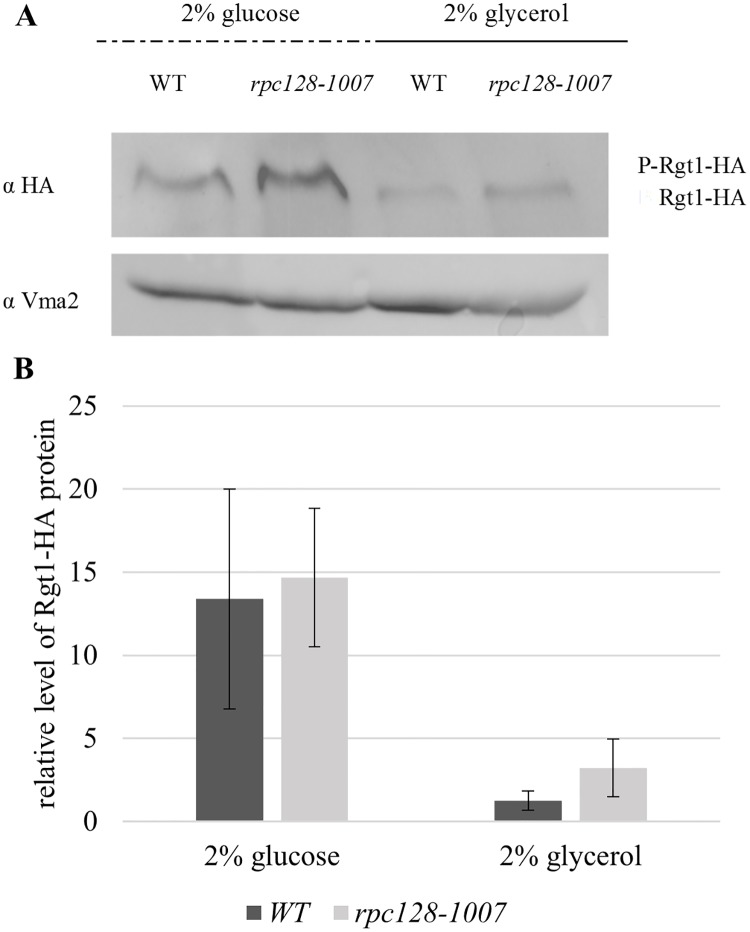
Cellular concentration and phosphorylation pattern of Rgt1 under repressing and activating growth conditions. The WT (MB159-4D) and *rpc128-100*7 strain expressing HA-tagged Rgt1 were grown to A_600_ ≈1.0 in 2% glucose or 2% glycerol-rich medium. Total cell protein extracts were subjected to SDS-PAGE and hybridized to anti-HA antibodies (A). The quantified relative level of Rgt1-HA (B) protein compared to Vma2 protein was quantified for three independent experiments in triplicate with standard deviations. The molecular weight (MW) of Rgt1-HA was ~144.7 kDa, and the MW of Vma2 was ~57.7 kDa.

Both in wild-type and *rpc128-1007* cells, the levels of Rgt1 protein were lower in the presence of a non-fermentable carbon source when compared with favorable growth conditions on glucose (Fig 7A and 7B). We did not observe any significant changes in the level of Rgt1 protein that were dependent on a strain used. However, anti-HA antibodies more efficiently precipitated chromatin fragments with Rgt1-3HA from cells carrying the *rpc128-1007* mutation (Fig 4C). The regulatory region of *HXT2*, which we divided into two fragments: one containing a single Rgt1 binding motif (downstream of UAS) and the other having two motifs in the UAS region, demonstrating a significantly increased occupancy by the transcription factor on DNA when compared with the reference strain. The degree of occupancy was proportional to the number of motifs available for Rgt1-specific binding in each fragment tested within the promoter region (Fig 4A).

The high occupancy of Rgt1 on the *HXT2* gene in the strain with elevated *HXT2* expression raises the possibility that Rgt1 may no longer acts as a repressor but rather as an activator of *HXT2* expression in cells with malfunctioning RNAP III. Of note, Rgt1 removal from DNA is not necessary for glucose induction of *HXT1* gene expression, which contains 8 Rgt1 binding motifs [40]. Moreover, Rgt1 is converted from a transcriptional repressor to an activator upon glucose-mediated phosphorylation [19]. Our data suggest that neither the removal of Rgt1 from DNA nor its phosphorylation status is a pre-requirement for *HXT2* accumulation in *rpc128-1007*. The conversion of Rgt1 into a functional activator may not occur in a phosphorylation-dependent manner in *rpc128-1007* cells grown on a non-fermentable carbon source.

### Tup1 contributes to the activation of *HXT2* transcriptional expression

The Ssn6-Tup1 protein complex is required for the repression of genes that are activated in response to alterations in growth conditions and cellular stresses, functioning as a mediator of glucose repression [87], [32]. It has been suggested that glucose regulates Rgt1 function by modulating Rgt1 interaction with Ssn6-Tup1, which is commonly referred as a corepressor complex [40] or as a repressor-activator complex [44]. Since in native yeast cells Rgt1 inhibits the transcription of the glucose transporter (*HXT*) genes in the absence of glucose, we wanted to further address why *HXT2* mRNA levels were elevated in *rpc128-1007* cells grown in glycerol medium by investigating the association of Tup1 with *HXT2* chromatin.

Using the same approach as previously, we created Tup1-3HA producing strains (Table 1), because Tup1 is a predominant component of the Ssn6-Tup1 complex. Tup1 associates with a variety of DNA-binding repressor proteins, among which Mig1 and Rgt1 directly recruit Tup1 to diverse sets of genes under various stress conditions and particular subsets of genes regulated by glucose [31], [32].

**Fig 8:**
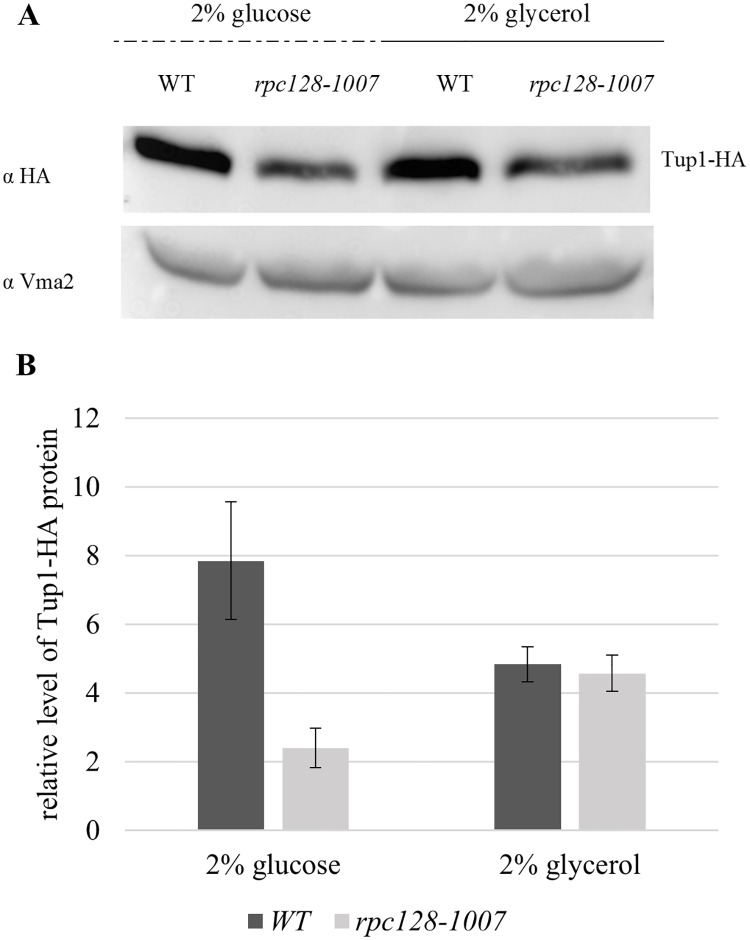
A low cellular concentration of Tup1 in the *rpc128-1007* mutant with low RNAP III activity. The WT (MB159-4D) and *rpc128-1007* strains expressing HA-tagged Tup1 proteins were grown to *A*_*600*_ ≈ 1.0 on 2% glucose or 2% glycerol-rich medium. Total cell protein extracts were subjected to SDS-PAGE and hybridized to anti-HA antibodies (A). The relative level of Tup1 (B) protein in comparison to Vma2 protein was quantified for three independent experiments conducted in triplicates with standard deviations. The molecular weight (MW) of Tup1 was ~89.88 kDa, and the Vma2 MW was ~57.7 kDa.

The abundance of Tup1 determined in *rpc128-1007* is presented in Fig 8(A) and 8(B). The result shows lower levels of Tup1 in r*pc128-1007* protein extracts under high-glucose conditions and no significant changes in Tup1 abundance in the absence of glucose. This may suggest that at least under high glucose conditions, even if Rgt1 is bound to the *HXT2* gene, the repressor complex may not be established due to the lower Tup1 occupancy.

In contrast to primary speculations, Tup1 was detected on the chromatin from the position -1 to -843, which encompasses the whole regulatory region of the *HXT2* promoter (Fig 4A). The association of Tup1 with the *HXT2* promoter was significantly higher in *rpc128-1007* than in the reference strain, particularly in cells grown in medium with glycerol (Fig 4D). It was especially enriched at the position shown herein to be bound by Mig1 and Rgt1, the known Tup1 recruiters. We postulate that, Rgt1/Ssn6-Tup1 corepressor complex may switch into an activator mode and thus accounts for the overexpression of *HXT2* in the strain with low RNAP III activity.

### Cells with defected RNAP III have an accurate perception of external glucose availability *via* Snf3/Rgt2-Rgt1 (SRR)

Mth1 blocks the protein kinase A—dependent phosphorylation of Rgt1 that impairs the ability of Rgt1 to interact with the Ssn6-Tup1 complex [30]. This phenomenon raises the possibility that Mth1 in *rpc128-1007* is not able to establish a stable repressor complex on the regulatory regions of the *HXT* genes.

Our observation of high Rgt1 occupancy on the *HXT2* promoter and elevated *HXT2* mRNAs, regardless of the carbon source, prompted us to investigate the effect of *rpc128-1007* on the cellular concentration of Mth1. Therefore, we constructed strains with chromosomally encoded HA epitope-tagged Mth1 (Table 1) and measured the level of Mth1-3HA in cells grown in the absence of glucose or in 2% glucose medium.

**Fig 9:**
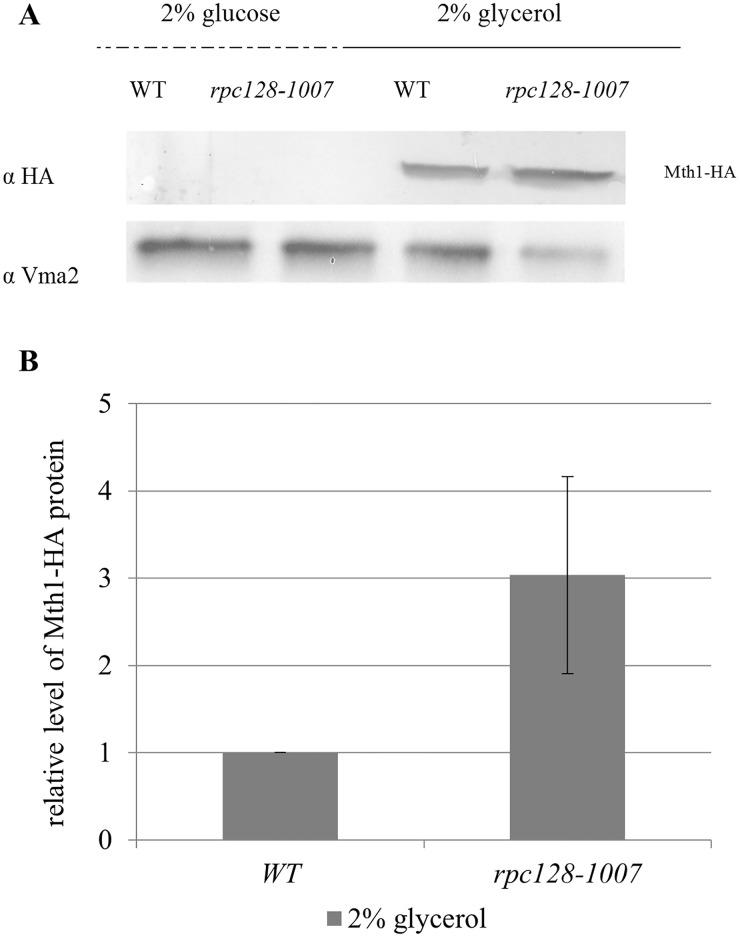
Increased stability of Mth1 in the *rpc128-1007* strain under non-fermentative growth conditions. The wild-type (MB159-4D) and *rpc128-1007* mutant strain expressing HA-tagged Mth1 were grown to A_600_ ≈ 1.0 in 2% glucose or 2% glycerol-rich medium. Proteins were extracted and immunoprecipitated with magnetic beads conjugated to mouse anti-HA antibodies. Mth1-HA was visualized with rabbit anti-HA tag antibodies. The relative level of Mth1 (B) protein in comparison to yeast Vma2 protein from four independent biological replicates. The molecular weight (MW) of Mth1 with the HA tag was ~56.28 kDa, and the MW Vma2 was ~57.7 kDa.

We found that glucose-dependent degradation of Mth1 was not impaired in the *rpc128-1007* strain. Mth1 degradation occurred in both strains, *rpc128-1007* and the control strain in the presence of high glucose concentrations (Fig 9A). Additionally, the observation of Mth1 being undetectable correlates with the decreased Rgt1 occupancy on the promoter region in the mutant and the reference strain. Cultivation of the strains on glycerol-rich medium led to an accumulation of Mth1 (Fig 9A and 9B). In *rpc128-1007*, we observed 3-fold higher Mth1 concentration, which may also explain the higher occupancy of Rgt1 on the *HXT2* promoter in *rpc128-1007* quantified in ChIP-qPCR assay.

In summary, we observed an increase in the stability of Mth1 in *rpc128-1007* on glycerol, which clearly promotes increased occupancy of Rgt1 on the *HXT2* promoter (Fig 4C).

Based on our understanding, the expression of *HXT2* is independent of Mth1 negative regulation of the Snf3/Rgt2-Rgt1 (SRR) glucose-sensing signal transduction pathway in the *rpc128-1007* background.

## Discussion

A high concentration of external glucose has been reported by several authors to reduce *HXT6/7* and *HXT2* transcription as a consequence of catabolite repression [73], [88], [3].

In this study, we observed that the *HXT6/7* transcript levels in RNAP III compromised strains, *rpc128-1007* and *maf1-Δ*, grown on glucose or glycerol-supplemented medium were negatively correlated and in generally different from the levels observed in wild-type yeast (Fig 2A). The *maf1-Δ*, in which transcription of the gluconeogenesis genes, *FBP1* and *PCK1*, is down regulated on glycerol, has reduced *HXT6/7* transcript levels, whereas *rpc128-1007*, which grows poorly in the presence of a high concentration of extracellular glucose (2%), reverses the defect and even exhibits enhanced *HXT6/7* transcription.

Regarding *HXT2*, the scenario was more complicated, because *HXT2* mRNA levels increased slightly in *maf1-Δ* on glucose and glycerol, whereas it was constitutive in *rpc128-1007* (fully derepressed) regardless of the fermentability of the carbon source (Fig 2B and 2C). Together, our primary data suggested that the mutant strains lost an environmental sensitivity for glucose. We presumed that glucose signaling may be perturbed in the *maf1-Δ* and in *rpc128-1007* mutants, but in this report, we excluded the possibility for *rpc128-1007* mutant providing a few lines of evidence.

In this study, we examined glucose signaling pathways in a RNAP III mutant, in which we observed not only the full induction of *HXT2* gene expression at high glucose concentrations but also after a shift to glycerol as the carbon source (Fig 2C).

This finding is particularly interesting because one of the most striking features of *rpc128-1007* is the severe growth defect on media containing high concentrations of glucose.

The transcription of some nutrient-regulated genes has been suggested to be affected by the cell growth rate. The transcription of such genes is the so called ‘growth rate signature’ or ‘poor growth’-specific gene transcriptional pattern [89], [16]. Zaman and Slattery [16], [90] independently proposed that the expression of genes that are directly dependent on the growth rate is determined by single-cell organism perception of the nutritional status of its surroundings. This perception usually corresponds to the actual nutrients availability. However, genetic manipulation may result in a discrepancy between what is perceived by the cell and the environmental availability of nutrients such as carbon sources [for details see [16]].

In this case study, we claim that the high, steady-state, mRNA levels of *HXT2* regardless of carbon source and its concentration in cells carrying a point mutation in RNAP III catalytic subunit C128 does not result simply from a poor growth rate (Fig 2C and 2D and S2 Fig), misperceived nutritional status of cell surrounding (Figs 4B, 5, 6 and 9), as a consequence of Snf3/Rgt2-Rgt1 (SRR) or Snf1-Mig1 glucose signaling impairment (Figs 4B and 4C, 5, 6 and 9) but result from other unknown yet but specific intracellular factor. This factor whose levels might change from optimal in wild type cells to unbalanced in cells exhibiting low RNAP III activity causes the Rgt1/Ssn6-Tup1 corepressor complex to switch into an activator mode on chromatin.

The growth rate is known to influence the cell cycle duration and expression of multiple genes, such ribosome biogenesis genes [89]. We provide evidence that the reason for “growth rate signature” can be narrowed down and described more specifically than originally though.

We included *rsp5-Δ* [SPT23^1-686^:URA3] mutant into this study and measured *HXT2* transcript levels in the strain by real-time-PCR. Rsp5 has been shown to be implicated in the activation of the plasma membrane H^+^-ATPase Pma1 by glucose [79]. Pma1 associates with Std1, which facilitates the repression of *HXT3* transcription and can aids in the establishment of Rgt2/Snf3-Rgt1 signaling pathway [26].

The *rsp5-Δ* [SPT23^1-686^:URA3] mutant, despite its growth rate reduction similar to *rpc128-1007*, did not display elevated *HXT2* transcript levels by several hundred fold as observed for *rpc128-1007* mutant cells. Thus, the failure in change of plasma membrane potential does not contribute to an accumulation of *HXT2* mRNA in *S*. *cerevisiae*. Thus, this is a specific feature of the strain with decreased RNAP III activity, and we were interested in elucidating this phenomenon in the context of functional or dysfunctional cell perception mechanisms.

As a consequence, we examined two major signaling pathways, the Snf1-Mig1 repression pathway and the Snf3/Rgt2-Rgt1 (SRR) induction pathway. The Snf3/Rgt2-Rgt1 (SRR) glucose signal transduction pathway is primarily dedicated to regulating the expression of *HXT* genes [20].

Since compromised RNAP III activity leads to decreased availability of tRNAs for protein synthesis, we wanted to explore whether the synthesis of repressor/activator proteins of affected gene would also be affected. No critical difference in regulatory proteins abundance was observed with an exemption of Mth1 which concentration apparently increased by 3 fold in *rpc128-1007* cells compared to the wild type isogenic strain (Fig 9).

We examined the pathway components, measured the concentrations of cellular proteins and their phosphorylation status and determined the occupancy of DNA regulatory regions to elucidate the cause of aberrant *HXT2* expression regulation in the *rpc128-1007* mutant.

We analyzed the whole, upstream regulatory region of *HXT2* using the *in vivo* chromatin immunoprecipitation assay with endogenous chromosomally encoded Rgt1-3HA, Mig1-3HA and Tup1-3HA, to define the association of major transcriptional regulators with *HXT2* chromatin.

We did not observe any difference in the Mig1 binding pattern to chromatin between the tested strains and in terms of glucose-dependent dephosphorylation of Mig1 repressor. In both strains, Mig1 occupancy of the *HXT2* promoter was equally abundant on high glucose and equally diminished on glycerol, which corresponded to its modification by phosphorylation (Fig 4A and 4B). We claim, that the slower migrating bands correspond to the phosphorylated form of Mig1 according to the online database BioGRID. Other PTM modifications have not been reported for Mig1 to date. Serine S222, S278, S311 and S381 in Mig1 amino acids chain are the PTM sites recognized by Snf1 [38], [91], [92], [93] and a single S264 site is targeted by cyclin-dependent kinase CDC28 associated with CLB2 cyclin, which among others regulates cell cycle and basal transcription in yeast [94]. The Fig 5A clearly shows an effect of multiple modification on Mig1 in cells grown on glycerol. Snf1 seems to acts as the predominant modifier for Mig1 however we do not exclude the involvement of CDC28 kinase in Mig1 modification process and in changes of cell cycle dependent signaling in *rpc128-1007*.

The phosphorylation pattern of Mig1 corresponded to the level of external glucose availability in both the *rpc128-1007* mutant and the wild-type control (Fig 5). On average, there was more Mig1 protein in cells grown on glycerol medium compared to favorable growth conditions, as observed for wild-type strains by other authors [95], [96].

Thus, we concluded that Snf1-Mig1 pathway signaling does not contribute to the change in *HXT2* expression in the *rpc128-1007* background when the cells are grown on 2% glucose. However, the cell perception *via* the active Snf1-Mig1 pathway was additionally confirmed.

We sought to determine the invertase activity (*SUC2* gene is regulated at the transcriptional level by Snf1 activity) in *rpc128-1007*. Enzymatic reactions carried out with freshly prepared extracts demonstrated no glucose repression relief in the *rpc128-1007* mutant (neither in *maf1-Δ*, whereas high invertase activity was confirmed for the *reg1-Δ* strain, which, has been reported to possess high transcriptional activity of *SUC2* in the presence of high glucose concentrations (Fig 6).

The above observations let us to conclude, that glucose repression signaling *via* the Snf1-Mig1 pathway does not play a role in increasing *HXT2* transcript levels in the *rpc128-1007* background. Thus, the increase in *HXT2* transcription is not simply due to the release of glucose repression but another mechanism accounts for glucose induction of *HXT2* in *rpc128-1007* cells.

Under glucose-deficient conditions, Rgt1 is typically recruited to the regulatory region of *HXT* genes. Rgt1 bound to DNA interacts with the Ssn6-Tup1 complex. In general, Tup1 is also recruited to many glucose-repressed genes by Rgt1, Mig1 and many other recruiting proteins [97], [98]. When glucose is scarce, Mth1 is responsible for maintaining *HXT2* repression by sustaining the interaction between Rgt1 and the Ssn6-Tup1 complex. Degradation of Mth1 is thought to inactivate the repressor function of Rgt1 and allow for its phosphorylation by PKA kinase downstream of Snf3 in the presence of glucose [21], [22]. Mth1, *via* an alteration of its own abundance, mirrors the extracellular glucose concentration. However, this model of gene induction lacks clarity, due to the fact that expression among the *HXT* gene family varies according to the wide range of glucose concentration.

Several reports have shown that the deletion of *MTH1* affects *HXT2* gene expression, which is induced as much as 400-fold by this mutation [24], [99]. Since we observed strong change (340-fold Fig 2C) in *HXT2* steady state mRNA levels in *rpc128-1007*, we examined the abundance of Mth1 in the mutant cells producing chromosomally encoded, a HA-tagged derivative of *MTH1* as the only functional copy of Mth1.

Mth1 was highly abundant in *rpc128-1007* and the reference strain during exposure to glycerol. On the other hand, our analysis revealed (Fig 9) that Mth1 protein was not detectable either in the reference strain nor in *rpc128-1007* on 2% glucose medium. This result is in the agreement with data provided by several literature sources showing that external glucose initiates Mth1 degradation *via* functional glucose signaling Snf3/Rgt2 –Rgt1 (SRR) pathway however it does not provide explanation to the observation of elevated *HXT2* mRNA transcripts in RNAP III compromised strain, both on 2% glucose and on glycerol.

In conclusion, our results indicate that Rgt1 in a complex with Tup1 is a predominant positive regulator of *HXT2* expression in the *rpc128-1007* mutant on non-fermentable carbon.

As indicated in Fig 4C in the *rpc128-1007* mutant there was observed an increased association of Rgt1 with chromatin in comparison to the isogenic reference strain grown in medium containing glycerol. The higher degree of Rgt1 occupancy on the *HXT2* gene in *rpc128-1007* was correlated with the greater quantified abundance of Mth1 (by 3-fold) (Fig 9). On the other hand, on glucose, the absence of Mth1 protein measured by anti-HA antibodies in both strains was correlated with an equally reduced abundance of Rgt1 on chromatin when high concentration of glucose was available. Nevertheless, Tup1 occupancy was significantly higher on the *HXT2* gene in *rpc128-1007* then in the reference strain regardless of the carbon source (Fig 4D).

mRNA metabolism is a very dynamic process. Steady state mRNA levels, are a mixture of effects on *de novo* synthesis and decay [100]. Knowing that the dynamic relationship between the activator/repressor proteins recruitment to the *HXT2* promoter has important implication for the mechanism of transcriptional activation and repression under different physiological conditions we aimed at investigating *de novo* synthesis of *HXT2*. The combination of chromatin immunoprecipitation (ChIP) and real-time PCR expression profiling methods, which we used, are widely used methods to determine the role of regulatory proteins and cooresponding gene expression profiles (also on large-scale genome-wide formats ChIP-ChIP and microarrays profiling) [67], [101]. We do not rule out the possibility that *HXT2* mRNA half-life might be extended in *rpc128-1007* mutant. However it seem to be contradictory, that repression of the *HXT2* transcription by significant enrichment of Tup1 corepressor on chromatin regardless carbon source availability, would be followed by mRNA stabilization.

Our results suggest a model, in which Rgt1 with Ssn6-Tup1 function as a transcriptional coactivator complex rather than a repressor complex, leading to the induction of *HXT2* in *rpc128-1007* (Figs 2C and 4D). In support of our conclusion, Rgt1 was previously found to be required to fully induce *HXT1* gene [102] and allows full expression of invertase in the absence of glucose [83]. In our study, the invertase activity was not elevated together with *HXT2*, raising speculation, that the regulatory mechanism superposed on *HXT2* in *rpc128-1007* is not a general mechanism governing the expression of the glucose-repressed genes.

We suspect, that no transcriptional factors other than Mig1 and Rgt1, recruit Tup1 to chromatin in *rpc128-1007*, because the highest occupancy of Tup1 was quantified by real-time PCR on cDNA fragments of amplified, marked 4 and 5 encompassing multiple binding sites of Mig1 and Rgt1 (Fig 4D). The binding motif for Nrg1, the global transcriptional repressor, is placed within the fragments 6 (position -843–683) and the upstream region (Fig 4A) in which not enrichment of Tup1 occupancy on the *HXT2* promoter is observed [103].

In this work, we further confirmed that Rgt1 phosphorylation influences the release of Rgt1 from the *HXT2* promoter in the presence of glucose (Figs 4C and 7A and 7B). The dissociation of Rgt1 has been noted by other authors as a general mechanism regulating *HXT1*, *HXT3* [9] and *HXK2* [104] genes induction [29], [30]. According to our observation dissociation of Rgt1 due to PTM modifications (Fig 7) is similar in *rpc128-1007* background and the control strain. This is unlikely to be the mechanism that contributes to the elevated steady-state *HXT2* transcripts in cells with low RNAP III activity. (Figs 2C and 4C). Thus, we propose that there must be an additional regulatory mechanism that modulates *HXT2* mRNA level in *rpc128-1007*. The enrichment of Tup1 protein abundance on *HXT2* regulatory region in *rpc128-1007* when compared to the isogenic wild-type strain both in the presence of glucose and glycerol suggest the involvement of the Ssn6-Tup1 complex in the process (Fig 4D and S3 Fig).

As noted by *Gancedo et al 2015* [83] in particular, the repression circuit that operates in the presence of high glucose levels does not require Rgt1 or Mth1, but involves Mig1, Hxk2, Ssn6 and Tup1 [105], [3]. We excluded that Mig1 plays a role in *HXT2* overexpression in the *rpc128-1007* background in the presence of high glucose levels. We did not tested *HXK2*, a component of the glucose repression signaling circuit, under the assumption that by estimating invertase activity we provide evidence that the Snf1-Mig1 pathway is not perturbed in the strain with low RNAP III activity.

However, the general transcriptional regulator Tup1 is a very good candidate as a potential regulator of *HXT2* based on our present data.

Tup1 chromatin-silencing transcriptional regulator in complex with Ssn6 regulates many genes in response to glucose, DNA damage, mating type and oxygen availability [32], [106]. It is recruited to gene promoters by a number of sequence-specific DNA binding and signaling pathway-specific repressor proteins [107], [108], [109], [110]. Tup1 interacts with histones H3 and H4 through repression domains present in its N-terminal of coiled-coil reach region [111]. Tup1 has been reported to function as both a corepressor and coactivator involved in the induction of gene expression. It is a transcriptional coactivator for Stp1/2-dependent transcription of amino acids transporter genes [112], *ARG1* and *ARG4* by enhancing the binding of Gcn4 to the UAS activation sequence [43]. It is responsible for the induction of osmotic-inducible promoters by switching from a repressing to an activating state that is regulated by the Hog1 MAP kinase pathway [46]. *GRE2* is another Tup1-represed gene whose transcription is activated by Ssn6-Tup1 and SAGA recruitment [46].

Several reports provide evidence that Tup1 remains associated with the promoters of target genes after activation. Whole-genome studies demonstrate that Tup1 binds to many glucose-repressed genes, even after the alleviation of glucose repression [46], [43], [113]. This is also a highly conserved protein in yeast, invertebrates and vertebrates. Human Tup1 homolog Groucho protein plays important role in neuronal development and is linked to several neuronal pathologies and Drosophila TLE protein is implicated in metabolic syndromes due pancreatic beta cell development [114], [115]. Thus, a better understanding of how the function of Tup1 is regulated in cells with perturbed RNAP III activity under different nutritional conditions will provide important new therapeutic opportunities.

We did not mutate the genes encoding Tup1 recruiter proteins, as shown by Hanon and coworkers [98] using a quantitative ChIP-ChIP analysis, for majority of Tup1 targets the elimination of a single recruit did not alter or eliminate Tup1 chromatin binding. This finding suggests that multiple, redundant DNA-binding proteins direct Tup1 to its target promoters. How the Ssn6-Tup1 complex receives signals from multiple signaling pathways, integrates those signals, and regulates gene expression accordingly is not known in details.

Our finding of a positive role for the Ssn6-Tup1 complex in *HXT2* transcription is a further evidence in support of the model in which specific metabolic signals may convert the Ssn6-Tup1 transcriptional corepressor to a coactivator of certain promoters and contribute to chromatin architecture and epigenetic status of genes regulation [116]. Tup1 interaction with PI(3,5)P2 lipid has been found as a triggering mechanism in *GAL1* gene induction [116], [117]. We have hypothesized that Tup1 associated with *HXT2* chromatin might be affected by PI(3,5)P2 lipid concentration, as Maf1, the regulator of RNAP III in a target of phosphoinositide 3-kinase (PI3K) signaling that negatively regulates oncogenesis and lipid metabolism in mice [118]. However examination of two mutant strains *fab1-Δ*,. that lacks the vacuolar membrane kinase generating PI(3,5)P2 and *vac14-Δ*, which synthesizes only modest amounts of the metabolite, demonstrates that *HXT2* steady state mRNA level, does not seem to be dependent on PI(3,5)P2 intracellular abundance (S4 Fig).

The alternative hypothesis for Tup1 specific mode switching would be its binding to differently posttranslationally modified transcriptional regulators and focusing signals from different signaling pathways, however we do not favor this possibility regarding Mig1 repressor.

Whole-organism scale analysis of metabolites and transcripts in yeast shows that intracellular changes in metabolite concentration are followed by changes the regulation of metabolic pathways through enzyme expression produced only *via* an orchestrated network of ribosome biogenesis and other components required for protein production. Thus, yeast modulate the level of protein synthesis accordingly to their metabolic requirements.

In theory, many intracellular factors such as the glucose flux, tRNA levels, other not yet tested Tup1 binding partners would contribute to the observed regulatory effects on *HXT2* expression in cells with low RNAP III activity.

The transport of glucose into the cell has been viewed as the rate-limiting step of *S*. *cerevisiae* glycolysis [72], [119], [120]. By examining the level of glucose uptake in yeast cells, we provide evidence that the glucose flux must be present in the mutants and the isogenic wild-type strain. A glucose flux was observed in *rpc128-1007* yeast cells, but it seemed to be less efficient in comparison to the wild-type control as measured by 2-NBD glucose uptake (Fig 3) however difficult to assess quantitively. The initial rate of 2-NBDG analog uptake is different from glucose and differs within species [121]. Yeast cells transport the fluorescent analog according to Michaelis-Menten kinetics with a capacity (V_max_) of 9.2 nM min^-1^mg dry weight^-1^ and a Michaelis constant (Km) of 6.1 mM. Thus, 2-NDBG glucose has the potential to measure activity of *HXT2* and *HXT6/7* facilitated diffusion system [122]. Glucose uptake depends not only on *HXT* expression but also on the posttranslational modification of Hxt proteins, which occurs in the presence of glucose [14], [72]. We cannot exclude the absence of differences in posttranslational modification of the transporters between the strains used in this study and whether the *rpc128-1007* background provides an intracellular environment for the synthesis of functional, properly modified and folded Hxt2 transporters. Elevated transcription of *HXT* genes does not always correspond to increased glucose uptake [14].

## Conclusions

**Fig 10:**
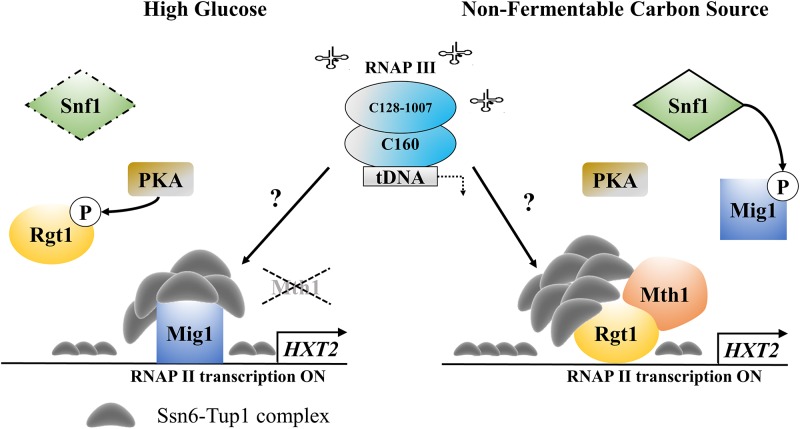
Low RNAP III activity caused by Gly1007Ala point mutation in C128 RNAP III subunit, correlates with transcription of *HXT2* gene by RNAP II, despite repressing conditions. Activation of the Snf1-dependent glucose repression and Snf3/Rgt2-Rgt1 (SRR) signaling pathways do not repress the *HXT2* transcription by RNAP II. We propose Ssn6-Tup1 complex as a *HXT2* transcription coactivator in *rpc128-1007*. Under high-glucose conditions, Mth1 degradation occurs. Rgt1, which is phosphorylated by PKA, dissociates from the *HXT2* promoter. Mig1, which is bound to the regulatory region, recruits Ssn6-Tup1. The complex transforms into a coactivator complex due to an unidentified intracellular signal and the expression of *HXT2* is induced. Under non-fermentable growth conditions in the strain with low RNAP III activity, the Snf3/Rgt2-Rgt1 (SRR) pathway transduces the signal for unfavorable external conditions to Mth1, preventing its degradation. The Rgt1 and Tup1 corepressor complex transforms into an activator complex and strongly induces *HXT2* expression.

Regarding *HXT2* expression, which was fully derepressed in the *rpc128-1007* mutant, we demonstrated that cellular perception of nutrient availability alone is not sufficient to establish the *HXT2* transcriptional pattern in yeast with compromised RNAP III activity (Fig 10). Thus, the well-known glucose signaling mechanisms involved in repression and induction of the gene become redundant.

It is likely that intracellular signal, either a proteins or a metabolite, serves as a superior regulatory element for Rgt1/Ssn6-Tup1 corepressor complex, which facilitates switching the complex into an activator mode and can wholly account for *HXT2* mRNA accumulation in the strain with low RNAP III activity. The concentration of this molecule is a gauge of the metabolic state of the cells with different RNAP III activity and does not allow the cells to complete transcription program in a glucose concentration-dependent manner.

## Supporting information

S1 Table Primers used to generate the deletion mutants and strains expressing the Mig1, Rgt1, Tup1 and Mth1 HA-tagged proteins. (DOCX)

S2 Table Plasmids used in the study. (DOCX)

S3 Table Oligonucleotide sequences of the primers used in the real-time PCR experiment. (DOCX)

S1 Fig Glucose depletion in yeast cultures. To determine the glucose concentration in media, aliquots were collected from cultures at regular intervals starting *A*_*600*_ = 0.2 and grown to *A*_*600*_ = 1.4. Data are expressed as the mean concentration, in mg of glucose in 1 ml of medium, obtained from at least three independent experiments conducted in triplicate. The standard deviation is expressed in mg of glucose in 1 ml of medium.
(TIF)

S2 Fig Growth curves of *rpc128-1007* and *rsp5-Δ [SPT23* ^*1–686*^:*URA3]*. Overnight yeast cultures grown in YPD medium supplemented with 2% glucose were diluted to A_600_ ≈ 0.2 and cultured for 12 h.
(TIF)

S3 Fig Tup1 occupancy profile on *HXT2* promoter in WT strain. The WT (MB159-4D) strain expressing HA-tagged Tup1 was grown in 2% glucose (YPD) or 2% glycerol (YPGly) rich medium. Crosslinked chromatin was immunoprecipitated with antibodies against the HA epitope, followed by real-time PCR. The signals are presented as the percent of the INPUT signal from three separate experiments with standard deviations. Numbers from 1 to 6 correspond to real-time PCR amplification products in the *HXT2* promoter region on the Fig 4A panel.
(TIF)

S4 Fig *HXT2* expression pattern in *fab1-Δ* and *vac14-Δ* mutant cells. Strains were cultured in rich medium (YP) supplemented with either 2% glucose or 2% glycerol. RNA was isolated when the culture reached A600 ≈ 1 and reversed-transcribed to cDNA. Isolated RNAs were examined by SYBR GREEN-based real-time PCR. The samples were normalized to two reference genes: *U2* spliceosomal RNA (*U2*) and small cytosolic RNA (*SCR1*). The bars represent ratios between the levels of respective mRNAs in the mutants and the control isogenic wild-type strain. The expression level in the WT strain (BY4741) was set as 1.0. The means ± standard deviations of the relative expression levels are shown. Asterisks (*) indicate p-values ≤ 0.05 determined by Chi Square Test.
(TIF)

## References

[1] Lin X, Zhang C-Y, Bai X-W, Song H-Y, Xiao D-G. Effects of MIG1, TUP1 and SSN6 deletion on maltose metabolism and leavening ability of baker’s yeast in lean dough. Microb Cell Factories. 2014;13:93. 10.1186/s12934-014-0093-4 PMC4094228 24993311

[2] Kim D, Song J-Y, Hahn J-S. Improvement of Glucose Uptake Rate and Production of Target Chemicals by Overexpressing Hexose Transporters and Transcriptional Activator Gcr1 in Saccharomyces cerevisiae. Appl Environ Microbiol. 2015 12 15;81(24):8392–401. doi: 10.1128/AEM.02056-15 26431967 10.1128/AEM.02056-15 PMC4644637

[3] Özcan S, Johnston M. Function and Regulation of Yeast Hexose Transporters. Microbiol Mol Biol Rev. 1999 9;63(3):554–69. 10477308 10.1128/mmbr.63.3.554-569.1999 PMC103746

[4] Schneper L, Düvel K, Broach JR. Sense and sensibility: nutritional response and signal integration in yeast. Curr Opin Microbiol. 2004 Grudzie;7(6):624–30. doi: 10.1016/j.mib.2004.10.002 15556035 10.1016/j.mib.2004.10.002

[5] Kolkman A, Daran-Lapujade P, Fullaondo A, Olsthoorn MMA, Pronk JT, Slijper M, et al Proteome analysis of yeast response to various nutrient limitations. Mol Syst Biol. 2006 5 16;2:2006.0026 doi: 10.1038/msb4100069 16738570 10.1038/msb4100069 PMC1681501

[6] Ronen M, Botstein D. Transcriptional response of steady-state yeast cultures to transient perturbations in carbon source. Proc Natl Acad Sci U S A. 2006 1 10;103(2):389–94. doi: 10.1073/pnas.0509978103 16381818 10.1073/pnas.0509978103 PMC1326188

[7] Rolland F, Winderickx J, Thevelein JM. Glucose-sensing and -signalling mechanisms in yeast. FEMS Yeast Res. 2002 Maj;2(2):183–201. 12702307 10.1111/j.1567-1364.2002.tb00084.x

[8] Boles E, Hollenberg CP. The molecular genetics of hexose transport in yeasts. FEMS Microbiol Rev. 1997 Sierpie;21(1):85–111. 9299703 10.1111/j.1574-6976.1997.tb00346.x

[9] Kim J-H, Johnston M. Two Glucose-sensing Pathways Converge on Rgt1 to Regulate Expression of Glucose Transporter Genes in Saccharomyces cerevisiae. J Biol Chem. 2006 8 9;281(36):26144–9. doi: 10.1074/jbc.M603636200 16844691 10.1074/jbc.M603636200

[10] Horák J. Regulations of sugar transporters: insights from yeast. Curr Genet. 2013 5 1;59(1–2):1–31. doi: 10.1007/s00294-013-0388-8 23455612 10.1007/s00294-013-0388-8

[11] Ozcan S, Dover J, Rosenwald AG, Wölfl S, Johnston M. Two glucose transporters in Saccharomyces cerevisiae are glucose sensors that generate a signal for induction of gene expression. Proc Natl Acad Sci U S A. 1996 10 29;93(22):12428–32. 8901598 10.1073/pnas.93.22.12428 PMC38008

[12] Coons DM, Vagnoli P, Bisson LF. The C-terminal Domain of Snf3p is Sufficient to Complement the Growth Defect of snf3 Null Mutations in Saccharomyces cerevisiae: SNF3 Functions in Glucose Recognition. Yeast. 1997 Stycze;13(1):9–20. doi: 10.1002/(SICI)1097-0061(199701)13:1<9::AID-YEA51>3.0.CO;2-U 9046082 10.1002/(SICI)1097-0061(199701)13:1<9::AID-YEA51>3.0.CO;2-U

[13] Maier A, Völker B, Boles E, Fuhrmann GF. Characterisation of glucose transport in Saccharomyces cerevisiae with plasma membrane vesicles (countertransport) and intact cells (initial uptake) with single Hxt1, Hxt2, Hxt3, Hxt4, Hxt6, Hxt7 or Gal2 transporters. FEMS Yeast Res. 2002 Grudzie;2(4):539–50. 12702270 10.1111/j.1567-1364.2002.tb00121.x

[14] Wendell DL, Bisson LF. Expression of high-affinity glucose transport protein Hxt2p of Saccharomyces cerevisiae is both repressed and induced by glucose and appears to be regulated posttranslationally. J Bacteriol. 1994 6;176(12):3730–7. 8206851 10.1128/jb.176.12.3730-3737.1994 PMC205562

[15] Liang H, Gaber RF. A novel signal transduction pathway in Saccharomyces cerevisiae defined by Snf3-regulated expression of HXT6. Mol Biol Cell. 1996 12;7(12):1953–66. 8970157 10.1091/mbc.7.12.1953 PMC276042

[16] Zaman S, Lippman SI, Schneper L, Slonim N, Broach JR. Glucose regulates transcription in yeast through a network of signaling pathways. Mol Syst Biol. 2009 2 17;5:245 doi: 10.1038/msb.2009.2 19225458 10.1038/msb.2009.2 PMC2657534

[17] Diderich JA, Schepper M, van Hoek P, Luttik MAH, van Dijken JP, Pronk JT, et al Glucose Uptake Kinetics and Transcription of HXTGenes in Chemostat Cultures of Saccharomyces cerevisiae. J Biol Chem. 1999 5 28;274(22):15350–9. 10336421 10.1074/jbc.274.22.15350

[18] Dietzel KL, Ramakrishnan V, Murphy EE, Bisson LF. MTH1 and RGT1 demonstrate combined haploinsufficiency in regulation of the hexose transporter genes in Saccharomyces cerevisiae. BMC Genet. 2012 12 12;13:107 doi: 10.1186/1471-2156-13-107 23234240 10.1186/1471-2156-13-107 PMC3564936

[19] Lakshmanan J, Mosley AL, Özcan S. Repression of transcription by Rgt1 in the absence of glucose requires Std1 and Mth1. Curr Genet. 2003 10 1;44(1):19–25. doi: 10.1007/s00294-003-0423-2 14508605 10.1007/s00294-003-0423-2

[20] Kaniak A, Xue Z, Macool D, Kim J-H, Johnston M. Regulatory Network Connecting Two Glucose Signal Transduction Pathways in Saccharomyces cerevisiae. Eukaryot Cell. 2004 1 2;3(1):221–31. doi: 10.1128/EC.3.1.221-231.2004 14871952 10.1128/EC.3.1.221-231.2004 PMC329515

[21] Ozcan S, Leong T, Johnston M. Rgt1p of Saccharomyces cerevisiae, a key regulator of glucose-induced genes, is both an activator and a repressor of transcription. Mol Cell Biol. 1996 1 11;16(11):6419–26. 8887670 10.1128/mcb.16.11.6419 PMC231643

[22] Moriya H, Johnston M. Glucose sensing and signaling in Saccharomyces cerevisiae through the Rgt2 glucose sensor and casein kinase I. Proc Natl Acad Sci U S A. 2004 10 2;101(6):1572–7. doi: 10.1073/pnas.0305901101 14755054 10.1073/pnas.0305901101 PMC341776

[23] Snowdon C, Johnston M. A novel role for yeast casein kinases in glucose sensing and signaling. Mol Biol Cell. 2016 11 1;27(21):3369–75. doi: 10.1091/mbc.E16-05-0342 27630263 10.1091/mbc.E16-05-0342 PMC5170868

[24] Lafuente MJ, Gancedo C, Jauniaux J-C, Gancedo JM. Mth1 receives the signal given by the glucose sensors Snf3 and Rgt2 in Saccharomyces cerevisiae‡. Mol Microbiol. 2000 Stycze;35(1):161–72. 10632886 10.1046/j.1365-2958.2000.01688.x

[25] Sabina J, Johnston M. Asymmetric Signal Transduction through Paralogs That Comprise a Genetic Switch for Sugar Sensing in Saccharomyces cerevisiae. J Biol Chem. 2009 10 23;284(43):29635–43. doi: 10.1074/jbc.M109.032102 19720826 10.1074/jbc.M109.032102 PMC2786032

[26] Brown JCS, Lindquist S. A heritable switch in carbon source utilization driven by an unusual yeast prion. Genes Dev. 2009 10 1;23(19):2320–32. doi: 10.1101/gad.1839109 19797769 10.1101/gad.1839109 PMC2758746

[27] Kim J-H, Polish J, Johnston M. Specificity and Regulation of DNA Binding by the Yeast Glucose Transporter Gene Repressor Rgt1. Mol Cell Biol. 2003 8;23(15):5208–16. doi: 10.1128/MCB.23.15.5208-5216.2003 12861007 10.1128/MCB.23.15.5208-5216.2003 PMC165726

[28] Polish JA, Kim J-H, Johnston M. How the Rgt1 Transcription Factor of Saccharomyces cerevisiae Is Regulated by Glucose. Genetics. 2005 2;169(2):583–94. doi: 10.1534/genetics.104.034512 15489524 10.1534/genetics.104.034512 PMC1449106

[29] Jouandot D, Roy A, Kim J-H. Functional dissection of the glucose signaling pathways that regulate the yeast glucose transporter gene (HXT) repressor Rgt1. J Cell Biochem. 2011 11;112(11):3268–75. doi: 10.1002/jcb.23253 21748783 10.1002/jcb.23253 PMC3341738

[30] Roy A, Shin YJ, Cho KH, Kim J-H. Mth1 regulates the interaction between the Rgt1 repressor and the Ssn6-Tup1 corepressor complex by modulating PKA-dependent phosphorylation of Rgt1. Mol Biol Cell. 2013 5 1;24(9):1493–503. doi: 10.1091/mbc.E13-01-0047 23468525 10.1091/mbc.E13-01-0047 PMC3639059

[31] Keleher CA, Redd MJ, Schultz J, Carlson M, Johnson AD. Ssn6-Tup1 is a general repressor of transcription in yeast. Cell. 1992 Luty;68(4):709–19. 1739976 10.1016/0092-8674(92)90146-4

[32] Smith RL, Johnson AD. Turning genes off by Ssn6–Tup1: a conserved system of transcriptional repression in eukaryotes. Trends Biochem Sci. 2000 Lipiec;25(7):325–30. 10871883 10.1016/s0968-0004(00)01592-9

[33] Sanz P, Alms GR, Haystead TAJ, Carlson M. Regulatory Interactions between the Reg1-Glc7 Protein Phosphatase and the Snf1 Protein Kinase. Mol Cell Biol. 2000 2;20(4):1321–8. 10648618 10.1128/mcb.20.4.1321-1328.2000 PMC85274

[34] Ahuatzi D, Riera A, Peláez R, Herrero P, Moreno F. Hxk2 Regulates the Phosphorylation State of Mig1 and Therefore Its Nucleocytoplasmic Distribution. J Biol Chem. 2007 2 16;282(7):4485–93. doi: 10.1074/jbc.M606854200 17178716 10.1074/jbc.M606854200

[35] Fernández-García P, Peláez R, Herrero P, Moreno F. Phosphorylation of Yeast Hexokinase 2 Regulates Its Nucleocytoplasmic Shuttling. J Biol Chem. 2012 7 12;287(50):42151–64. doi: 10.1074/jbc.M112.401679 23066030 10.1074/jbc.M112.401679 PMC3516761

[36] Vega M, Riera A, Fernández-Cid A, Herrero P, Moreno F. Hexokinase 2 Is an Intracellular Glucose Sensor of Yeast Cells That Maintains the Structure and Activity of Mig1 Protein Repressor Complex. J Biol Chem. 2016 1 4;291(14):7267–85. doi: 10.1074/jbc.M115.711408 26865637 10.1074/jbc.M115.711408 PMC4817161

[37] Hardie DG, Ross FA, Hawley SA. AMPK: a nutrient and energy sensor that maintains energy homeostasis. Nat Rev Mol Cell Biol. 2012 Kwiecie;13(4):251–62. doi: 10.1038/nrm3311 22436748 10.1038/nrm3311 PMC5726489

[38] Treitel MA, Kuchin S, Carlson M. Snf1 Protein Kinase Regulates Phosphorylation of the Mig1 Repressor in Saccharomyces cerevisiae. Mol Cell Biol. 1998 11;18(11):6273–80. 9774644 10.1128/mcb.18.11.6273 PMC109214

[39] Broach JR. Nutritional Control of Growth and Development in Yeast. Genetics. 2012 9 1;192(1):73–105. doi: 10.1534/genetics.111.135731 22964838 10.1534/genetics.111.135731 PMC3430547

[40] Roy A, Jouandot D, Cho KH, Kim J-H. Understanding the mechanism of glucose-induced relief of Rgt1-mediated repression in yeast. FEBS Open Bio. 2014 1 3;4:105–11. doi: 10.1016/j.fob.2013.12.004 24490134 10.1016/j.fob.2013.12.004 PMC3907687

[41] van Oevelen CJC, van Teeffelen HAAM, van Werven FJ, Timmers HTM. Snf1p-dependent Spt-Ada-Gcn5-acetyltransferase (SAGA) Recruitment and Chromatin Remodeling Activities on the HXT2 and HXT4 Promoters. J Biol Chem. 2006 2 17;281(7):4523–31. doi: 10.1074/jbc.M509330200 16368692 10.1074/jbc.M509330200

[42] Davie JK, Edmondson DG, Coco CB, Dent SYR. Tup1-Ssn6 Interacts with Multiple Class I Histone Deacetylases in Vivo. J Biol Chem. 2003 12 12;278(50):50158–62. doi: 10.1074/jbc.M309753200 14525981 10.1074/jbc.M309753200

[43] Kim S, Swanson MJ, Qiu H, Govind CK, Hinnebusch AG. Activator Gcn4p and Cyc8p/Tup1p Are Interdependent for Promoter Occupancy at ARG1 In Vivo. Mol Cell Biol. 2005 12 15;25(24):11171–83. doi: 10.1128/MCB.25.24.11171-11183.2005 16314536 10.1128/MCB.25.24.11171-11183.2005 PMC1316967

[44] Wong KH, Struhl K. The Cyc8–Tup1 complex inhibits transcription primarily by masking the activation domain of the recruiting protein. Genes Dev. 2011 12 1;25(23):2525–39. doi: 10.1101/gad.179275.111 22156212 10.1101/gad.179275.111 PMC3243062

[45] Papamichos-Chronakis M, Petrakis T, Ktistaki E, Topalidou I, Tzamarias D. Cti6, a PHD Domain Protein, Bridges the Cyc8-Tup1 Corepressor and the SAGA Coactivator to Overcome Repression at GAL1. Mol Cell. 2002 Czerwiec;9(6):1297–305. 12086626 10.1016/s1097-2765(02)00545-2

[46] Proft M, Struhl K. Hog1 Kinase Converts the Sko1-Cyc8-Tup1 Repressor Complex into an Activator that Recruits SAGA and SWI/SNF in Response to Osmotic Stress. Mol Cell. 2002 Czerwiec;9(6):1307–17. 12086627 10.1016/s1097-2765(02)00557-9

[47] Kresnowati MTAP, van Winden WA, Almering MJH, ten Pierick A, Ras C, Knijnenburg TA, et al When transcriptome meets metabolome: fast cellular responses of yeast to sudden relief of glucose limitation. Mol Syst Biol. 2006 9 12;2:49 doi: 10.1038/msb4100083 16969341 10.1038/msb4100083 PMC1681515

[48] Ghavidel A, Kislinger T, Pogoutse O, Sopko R, Jurisica I, Emili A. Impaired tRNA Nuclear Export Links DNA Damage and Cell-Cycle Checkpoint. Cell. 2007 Listopad;131(5):915–26. doi: 10.1016/j.cell.2007.09.042 18045534 10.1016/j.cell.2007.09.042

[49] Cieśla M, Towpik J, Graczyk D, Oficjalska-Pham D, Harismendy O, Suleau A, et al Maf1 Is Involved in Coupling Carbon Metabolism to RNA Polymerase III Transcription. Mol Cell Biol. 2007 11;27(21):7693–702. doi: 10.1128/MCB.01051-07 17785443 10.1128/MCB.01051-07 PMC2169064

[50] Vannini A, Ringel R, Kusser AG, Berninghausen O, Kassavetis GA, Cramer P. Molecular Basis of RNA Polymerase III Transcription Repression by Maf1. Cell. 2010 Październik;143(1):59–70. doi: 10.1016/j.cell.2010.09.002 20887893 10.1016/j.cell.2010.09.002

[51] Boguta M, Graczyk D. RNA polymerase III under control: repression and de-repression. Trends Biochem Sci. 2011 Wrzesie;36(9):451–6. doi: 10.1016/j.tibs.2011.06.008 21816617 10.1016/j.tibs.2011.06.008

[52] Pluta K, Lefebvre O, Martin NC, Smagowicz WJ, Stanford DR, Ellis SR, et al Maf1p, a Negative Effector of RNA Polymerase III in Saccharomyces cerevisiae. Mol Cell Biol. 2001 8;21(15):5031–40. doi: 10.1128/MCB.21.15.5031-5040.2001 11438659 10.1128/MCB.21.15.5031-5040.2001 PMC87229

[53] Upadhya R, Lee J, Willis IM. Maf1 Is an Essential Mediator of Diverse Signals that Repress RNA Polymerase III Transcription. Mol Cell. 2002 Grudzie;10(6):1489–94. 12504022 10.1016/s1097-2765(02)00787-6

[54] Reina JH, Azzouz TN, Hernandez N. Maf1, a New Player in the Regulation of Human RNA Polymerase III Transcription. PLOS ONE. 2006 12 27;1(1):e134. 17205138 10.1371/journal.pone.0000134 PMC1762419

[55] Morawiec E, Wichtowska D, Graczyk D, Conesa C, Lefebvre O, Boguta M. Maf1, repressor of tRNA transcription, is involved in the control of gluconeogenetic genes in Saccharomyces cerevisiae. Gene. 2013 Sierpie;526(1):16–22. doi: 10.1016/j.gene.2013.04.055 23657116 10.1016/j.gene.2013.04.055

[56] Cieśla M, Makała E, Płonka M, Bazan R, Gewartowski K, Dziembowski A, et al Rbs1, a New Protein Implicated in RNA Polymerase III Biogenesis in Yeast Saccharomyces cerevisiae. Mol Cell Biol. 2015 4;35(7):1169–81. doi: 10.1128/MCB.01230-14 25605335 10.1128/MCB.01230-14 PMC4355530

[57] Ferreira C, Lucas C. Glucose repression over Saccharomyces cerevisiae glycerol/H+ symporter gene STL1 is overcome by high temperature. FEBS Lett. 2007 Maj;581(9):1923–7. doi: 10.1016/j.febslet.2007.03.086 17434487 10.1016/j.febslet.2007.03.086

[58] Kwapisz M, Smagowicz WJ, Oficjalska D, Hatin I, Rousset J-P, Żołądek T, et al Up-regulation of tRNA biosynthesis affects translational readthrough in maf1-Δ mutant of Saccharomyces cerevisiae. Curr Genet. 2002 12 1;42(3):147–52. doi: 10.1007/s00294-002-0342-7 12491008 10.1007/s00294-002-0342-7

[59] Gietz RD, Schiestl RH. Large-scale high-efficiency yeast transformation using the LiAc/SS carrier DNA/PEG method. Nat Protoc. 2007 Stycze;2(1):38–41. doi: 10.1038/nprot.2007.15 17401336 10.1038/nprot.2007.15

[60] Longtine MS, Mckenzie A III, Demarini DJ, Shah NG, Wach A, Brachat A, et al Additional modules for versatile and economical PCR-based gene deletion and modification in Saccharomyces cerevisiae. Yeast. 1998 Lipiec;14(10):953–61. doi: 10.1002/(SICI)1097-0061(199807)14:10<953::AID-YEA293>3.0.CO;2-U 9717241 10.1002/(SICI)1097-0061(199807)14:10<953::AID-YEA293>3.0.CO;2-U

[61] Sherman F. Getting started with yeast. Methods Enzymol. 2002 Stycze;350:3–41. 12073320 10.1016/s0076-6879(02)50954-x

[62] Schmitt ME, Brown TA, Trumpower BL. A rapid and simple method for preparation of RNA from Saccharomyces cerevisiae. Nucleic Acids Res. 1990 5 25;18(10):3091–2. 2190191 10.1093/nar/18.10.3091 PMC330876

[63] Vandesompele J, De Preter K, Pattyn F, Poppe B, Van Roy N, De Paepe A, et al Accurate normalization of real-time quantitative RT-PCR data by geometric averaging of multiple internal control genes. Genome Biol. 2002;3(7):research0034.1–research0034.11. 12184808 10.1186/gb-2002-3-7-research0034 PMC126239

[64] Andersen CL, Jensen JL, Ørntoft TF. Normalization of Real-Time Quantitative Reverse Transcription-PCR Data: A Model-Based Variance Estimation Approach to Identify Genes Suited for Normalization, Applied to Bladder and Colon Cancer Data Sets. Cancer Res. 2004 8 1;64(15):5245–50. doi: 10.1158/0008-5472.CAN-04-0496 15289330 10.1158/0008-5472.CAN-04-0496

[65] Livak KJ, Schmittgen TD. Analysis of Relative Gene Expression Data Using Real-Time Quantitative PCR and the 2−ΔΔCT Method. Methods. 2001 Grudzie;25(4):402–8. doi: 10.1006/meth.2001.1262 11846609 10.1006/meth.2001.1262

[66] Simpson-Lavy KJ, Bronstein A, Kupiec M, Johnston M. Cross-talk between carbon metabolism and the DNA damage response in S. cerevisiae. Cell Rep. 2015 9 22;12(11):1865–75. doi: 10.1016/j.celrep.2015.08.025 26344768 10.1016/j.celrep.2015.08.025 PMC4581987

[67] Ren B, Robert F, Wyrick JJ, Aparicio O, Jennings EG, Simon I, et al Genome-Wide Location and Function of DNA Binding Proteins. Science. 2000 12 22;290(5500):2306–9. doi: 10.1126/science.290.5500.2306 11125145 10.1126/science.290.5500.2306

[68] Lin X, Tirichine L, Bowler C. Protocol: Chromatin immunoprecipitation (ChIP) methodology to investigate histone modifications in two model diatom species. Plant Methods. 2012;8:48 doi: 10.1186/1746-4811-8-48 23217141 10.1186/1746-4811-8-48 PMC3546051

[69] Achilles J, Müller S, Bley T, Babel W. Affinity of single S. cerevisiae cells to 2-NBDglucose under changing substrate concentrations. Cytometry A. 2004 Wrzesie;61A(1):88–98. 10.1002/cyto.a.20035 15351993

[70] Silveira MCF, Carvajal E, Bon EPS. Assay forin VivoYeast Invertase Activity Using NaF. Anal Biochem. 1996 Czerwiec;238(1):26–8. doi: 10.1006/abio.1996.0244 8660580 10.1006/abio.1996.0244

[71] Bradford MM. A rapid and sensitive method for the quantitation of microgram quantities of protein utilizing the principle of protein-dye binding. Anal Biochem. 1976 Maj;72(1):248–54. 942051 10.1016/0003-2697(76)90527-3

[72] Kruckeberg AL, Ye L, Berden JA, van Dam K. Functional expression, quantification and cellular localization of the Hxt2 hexose transporter of Saccharomyces cerevisiae tagged with the green fluorescent protein. Biochem J. 1999 4 15;339(Pt 2):299–307. 10191260 PMC1220158

[73] Ozcan S, Johnston M. Three different regulatory mechanisms enable yeast hexose transporter (HXT) genes to be induced by different levels of glucose. Mol Cell Biol. 1995 3;15(3):1564–72. 7862149 10.1128/mcb.15.3.1564 PMC230380

[74] Reifenberger E, Boles E, Ciriacy M. Kinetic Characterization of Individual Hexose Transporters of Saccharomyces Cerevisiae and their Relation to the Triggering Mechanisms of Glucose Repression. Eur J Biochem. 1997 Kwiecie;245(2):324–33. 9151960 10.1111/j.1432-1033.1997.00324.x

[75] Ingham RJ, Gish G, Pawson T. The Nedd4 family of E3 ubiquitin ligases: functional diversity within a common modular architecture. Oncogene. 2004;23(11):1972–84. doi: 10.1038/sj.onc.1207436 15021885 10.1038/sj.onc.1207436

[76] Snowdon C, van der Merwe G. Regulation of Hxt3 and Hxt7 Turnover Converges on the Vid30 Complex and Requires Inactivation of the Ras/cAMP/PKA Pathway in Saccharomyces cerevisiae. PLOS ONE. 2012 12 5;7(12):e50458 doi: 10.1371/journal.pone.0050458 23227176 10.1371/journal.pone.0050458 PMC3515616

[77] MacGurn JA, Hsu P-C, Emr SD. Ubiquitin and Membrane Protein Turnover: From Cradle to Grave. Annu Rev Biochem. 2012;81(1):231–59. 22404628 10.1146/annurev-biochem-060210-093619

[78] O’Donnell AF, McCartney RR, Chandrashekarappa DG, Zhang BB, Thorner J, Schmidt MC. 2-Deoxyglucose Impairs Saccharomyces cerevisiae Growth by Stimulating Snf1-Regulated and α-Arrestin-Mediated Trafficking of Hexose Transporters 1 and 3. Mol Cell Biol. 2015 3;35(6):939–55. doi: 10.1128/MCB.01183-14 25547292 10.1128/MCB.01183-14 PMC4333089

[79] K J, T A, G M, Z T. The growth of mdp1/rsp5 mutants of Saccharomyces cerevisiae is affected by mutations in the ATP-binding domain of the plasma membrane H+ -ATPase. Gene. 2000 1;242(1–2):133–40. 10721705 10.1016/s0378-1119(99)00535-1

[80] Weinhandl K, Winkler M, Glieder A, Camattari A. Carbon source dependent promoters in yeasts. Microb Cell Factories. 2014;13(1):5. 10.1186/1475-2859-13-5 PMC3897899 24401081

[81] Wang J, Sirenko O, Needleman R. Genomic Footprinting of Mig1p in the MAL62 Promoter binding is dependent upon carbon source and competitive with the Mal63p activator. J Biol Chem. 1997 2 14;272(7):4613–22. 9020190 10.1074/jbc.272.7.4613

[82] Mierzejewska J, Chreptowicz K. Lack of Maf1 enhances pyruvate kinase activity and fermentative metabolism while influencing lipid homeostasis in Saccharomyces cerevisiae. FEBS Lett. 2016 Stycze;590(1):93–100. doi: 10.1002/1873-3468.12033 26787463 10.1002/1873-3468.12033

[83] Jm G, Cl F, C G. The repressor Rgt1 and the cAMP-dependent protein kinases control the expression of the SUC2 gene in Saccharomyces cerevisiae. Biochim Biophys Acta. 2015 7;1850(7):1362–7. doi: 10.1016/j.bbagen.2015.03.006 25810078 10.1016/j.bbagen.2015.03.006

[84] Bu Y, Schmidt MC. Identification of cis-acting elements in the SUC2 promoter of Saccharomyces cerevisiae required for activation of transcription. Nucleic Acids Res. 1998 2 1;26(4):1002–9. 9461460 10.1093/nar/26.4.1002 PMC147334

[85] Ozcan S, Dover J, Johnston M. Glucose sensing and signaling by two glucose receptors in the yeast Saccharomyces cerevisiae. EMBO J. 1998 5 1;17(9):2566–73. doi: 10.1093/emboj/17.9.2566 9564039 10.1093/emboj/17.9.2566 PMC1170598

[86] Kim J-H. DNA-binding properties of the yeast Rgt1 repressor. Biochimie. 2009 2;91(2):300–3. doi: 10.1016/j.biochi.2008.09.002 18950675 10.1016/j.biochi.2008.09.002 PMC2859070

[87] Williams FE, Trumbly RJ. Characterization of TUP1, a mediator of glucose repression in Saccharomyces cerevisiae. Mol Cell Biol. 1990 12;10(12):6500–11. 2247069 10.1128/mcb.10.12.6500-6511.1990 PMC362927

[88] Gancedo JM. Yeast Carbon Catabolite Repression. Microbiol Mol Biol Rev. 1998 6;62(2):334–61. 9618445 10.1128/mmbr.62.2.334-361.1998 PMC98918

[89] Brauer MJ, Huttenhower C, Airoldi EM, Rosenstein R, Matese JC, Gresham D, et al Coordination of Growth Rate, Cell Cycle, Stress Response, and Metabolic Activity in Yeast. Mol Biol Cell. 2008 1;19(1):352–67. doi: 10.1091/mbc.E07-08-0779 17959824 10.1091/mbc.E07-08-0779 PMC2174172

[90] Slattery MG, Liko D, Heideman W. Protein Kinase A, TOR, and Glucose Transport Control the Response to Nutrient Repletion in Saccharomyces cerevisiae. Eukaryot Cell. 2008 1 2;7(2):358–67. doi: 10.1128/EC.00334-07 18156291 10.1128/EC.00334-07 PMC2238170

[91] Östling J, Ronne H. Negative control of the Mig1p repressor by Snf1p-dependent phosphorylation in the absence of glucose. Eur J Biochem. 1998 Luty;252(1):162–8. 9523726 10.1046/j.1432-1327.1998.2520162.x

[92] DeVit MJ, Johnston M. The nuclear exportin Msn5 is required for nuclear export of the Mig1 glucose repressor of Saccharomyces cerevisiae. Curr Biol. 1999 Listopad;9(21):1231–41. 10556086 10.1016/s0960-9822(99)80503-x

[93] Smith FC, Davies SP, Wilson WA, Carling D, Hardie DG. The SNF1 kinase complex from Saccharomyces cerevisiae phosphorylates the transcriptional repressor protein Mig1p in vitro at four sites within or near regulatory domain 1. FEBS Lett. 1999 Czerwiec;453(1–2):219–23. 10403407 10.1016/s0014-5793(99)00725-5

[94] Holt LJ, Tuch BB, Villén J, Johnson AD, Gygi SP, Morgan DO. Global Analysis of Cdk1 Substrate Phosphorylation Sites Provides Insights into Evolution. Science. 2009 9 25;325(5948):1682–6. doi: 10.1126/science.1172867 19779198 10.1126/science.1172867 PMC2813701

[95] Wollman AJ, Leake M. Millisecond single-molecule localization microscopy combined with convolution analysis and automated image segmentation to determine protein concentrations in complexly structured, functional cells, one cell at a time. Faraday Discuss. 2015;184(0):401–24. 26419209 10.1039/c5fd00077g

[96] McCartney RR, Chandrashekarappa DG, Zhang BB, Schmidt MC. Genetic Analysis of Resistance and Sensitivity to 2-Deoxyglucose in Saccharomyces cerevisiae. Genetics. 2014 10;198(2):635–46. doi: 10.1534/genetics.114.169060 25116136 10.1534/genetics.114.169060 PMC4196618

[97] Nehlin JO, Carlberg M, Ronne H. Control of yeast GAL genes by MIG1 repressor: a transcriptional cascade in the glucose response. EMBO J. 1991 11;10(11):3373–7. 1915298 10.1002/j.1460-2075.1991.tb04901.x PMC453065

[98] Hanlon SE, Rizzo JM, Tatomer DC, Lieb JD, Buck MJ. The Stress Response Factors Yap6, Cin5, Phd1, and Skn7 Direct Targeting of the Conserved Co-Repressor Tup1-Ssn6 in S. cerevisiae. PLoS ONE [Internet]. 2011 4 28 [cited 2017 May 9];6(4). Available from: http://www.ncbi.nlm.nih.gov/pmc/articles/PMC3084262/10.1371/journal.pone.0019060 PMC3084262 21552514

[99] Schulte F, Wieczorke R, Hollenberg CP, Boles E. The HTR1 Gene Is a Dominant Negative Mutant Allele of MTH1 and Blocks Snf3- and Rgt2-Dependent Glucose Signaling in Yeast. J Bacteriol. 2000 1 15;182(2):540–2. 10629208 10.1128/jb.182.2.540-542.2000 PMC94311

[100] Haimovich G, Medina DA, Causse SZ, Garber M, Millán-Zambrano G, Barkai O, et al Gene Expression Is Circular: Factors for mRNA Degradation Also Foster mRNA Synthesis. Cell. 2013 Maj;153(5):1000–11. doi: 10.1016/j.cell.2013.05.012 23706738 10.1016/j.cell.2013.05.012

[101] Kim J, Iyer VR. Global Role of TATA Box-Binding Protein Recruitment to Promoters in Mediating Gene Expression Profiles. Mol Cell Biol. 2004 9 15;24(18):8104–12. doi: 10.1128/MCB.24.18.8104-8112.2004 15340072 10.1128/MCB.24.18.8104-8112.2004 PMC515063

[102] Mosley AL, Lakshmanan J, Aryal BK, Özcan S. Glucose-mediated Phosphorylation Converts the Transcription Factor Rgt1 from a Repressor to an Activator. J Biol Chem. 2003 3 21;278(12):10322–7. doi: 10.1074/jbc.M212802200 12527758 10.1074/jbc.M212802200

[103] Lee SB, Kang HS, Kim T. Nrg1 functions as a global transcriptional repressor of glucose-repressed genes through its direct binding to the specific promoter regions. Biochem Biophys Res Commun. 2013 Październik;439(4):501–5. doi: 10.1016/j.bbrc.2013.09.015 24025681 10.1016/j.bbrc.2013.09.015

[104] Palomino A, Herrero P, Moreno F. Tpk3 and Snf1 protein kinases regulate Rgt1 association with Saccharomyces cerevisiae HXK2 promoter. Nucleic Acids Res. 2006;34(5):1427–38. doi: 10.1093/nar/gkl028 16528100 10.1093/nar/gkl028 PMC1401511

[105] Trumbly RJ. Glucose repression in the yeast Saccharomyces cerevisiae. Mol Microbiol. 1992 Stycze;6(1):15–21. 1310793 10.1111/j.1365-2958.1992.tb00832.x

[106] Matsumura H, Kusaka N, Nakamura T, Tanaka N, Sagegami K, Uegaki K, et al Crystal Structure of the N-terminal Domain of the Yeast General Corepressor Tup1p and Its Functional Implications. J Biol Chem. 2012 3 8;287(32):26528–38. doi: 10.1074/jbc.M112.369652 22707714 10.1074/jbc.M112.369652 PMC3410994

[107] Balasubramanian B, Lowry CV, Zitomer RS. The Rox1 repressor of the Saccharomyces cerevisiae hypoxic genes is a specific DNA-binding protein with a high-mobility-group motif. Mol Cell Biol. 1993 10;13(10):6071–8. 8413209 10.1128/mcb.13.10.6071-6078.1993 PMC364667

[108] Komachi K, Redd MJ, Johnson AD. The WD repeats of Tup1 interact with the homeo domain protein alpha 2. Genes Dev. 1994 1 12;8(23):2857–67. 7995523 10.1101/gad.8.23.2857

[109] Huang M, Zhou Z, Elledge SJ. The DNA Replication and Damage Checkpoint Pathways Induce Transcription by Inhibition of the Crt1 Repressor. Cell. 1998 Wrzesie;94(5):595–605. 9741624 10.1016/s0092-8674(00)81601-3

[110] C Rs, T D. Sfl1 functions via the co-repressor Ssn6-Tup1 and the cAMP-dependent protein kinase Tpk2. J Mol Biol. 2001 6;309(5):1007–15. doi: 10.1006/jmbi.2001.4742 11399075 10.1006/jmbi.2001.4742

[111] Edmondson DG, Smith MM, Roth SY. Repression domain of the yeast global repressor Tup1 interacts directly with histones H3 and H4. Genes Dev. 1996 5 15;10(10):1247–59. 8675011 10.1101/gad.10.10.1247

[112] Tanaka N, Mukai Y. Yeast Cyc8p and Tup1p proteins function as coactivators for transcription of Stp1/2p-dependent amino acid transporter genes. Biochem Biophys Res Commun. 2015 Grudzie;468(1–2):32–8. doi: 10.1016/j.bbrc.2015.11.001 26546823 10.1016/j.bbrc.2015.11.001

[113] Buck MJ, Lieb JD. A chromatin-mediated mechanism for specification of conditional transcription factor targets. Nat Genet. 2006 12;38(12):1446–51. doi: 10.1038/ng1917 17099712 10.1038/ng1917 PMC2756100

[114] Zhang Y, Qi H, Taylor R, Xu W, Liu LF, Jin S. The role of autophagy in mitochondria maintenance: characterization of mitochondrial functions in autophagy-deficient S. cerevisiae strains. Autophagy. 2007 8;3(4):337–46. 17404498 10.4161/auto.4127

[115] Metzger DE, Liu C, Ziaie AS, Naji A, Zaret KS. Grg3/TLE3 and Grg1/TLE1 Induce Monohormonal Pancreatic β-Cells While Repressing α-Cell Functions. Diabetes. 2014 5;63(5):1804–16. doi: 10.2337/db13-0867 24487024 10.2337/db13-0867 PMC3994953

[116] Conlan RS, Gounalaki N, Hatzis P, Tzamarias D. The Tup1-Cyc8 Protein Complex Can Shift from a Transcriptional Co-repressor to a Transcriptional Co-activator. J Biol Chem. 1999 1 1;274(1):205–10. 9867831 10.1074/jbc.274.1.205

[117] Han B-K, Emr SD. Phosphoinositide [PI(3,5)P2] lipid-dependent regulation of the general transcriptional regulator Tup1. Genes Dev. 2011 5 1;25(9):984–95. doi: 10.1101/gad.1998611 21536737 10.1101/gad.1998611 PMC3084031

[118] Palian BM, Rohira AD, Johnson SAS, He L, Zheng N, Dubeau L, et al Maf1 Is a Novel Target of PTEN and PI3K Signaling That Negatively Regulates Oncogenesis and Lipid Metabolism. PLOS Genet. 2014 12 11;10(12):e1004789 doi: 10.1371/journal.pgen.1004789 25502566 10.1371/journal.pgen.1004789 PMC4263377

[119] Reijenga KA, Snoep JL, Diderich JA, van Verseveld HW, Westerhoff HV, Teusink B. Control of glycolytic dynamics by hexose transport in Saccharomyces cerevisiae. Biophys J. 2001 2;80(2):626–34. doi: 10.1016/S0006-3495(01)76043-2 11159431 10.1016/S0006-3495(01)76043-2 PMC1301262

[120] Ye L, Berden JA, van Dam K, Kruckeberg AL. Expression and activity of the Hxt7 high-affinity hexose transporter of Saccharomyces cerevisiae. Yeast. 2001 Wrzesie;18(13):1257–67. doi: 10.1002/yea.771 11561293 10.1002/yea.771

[121] Tao J, Diaz RK, Teixeira CRV, Hackmann TJ. Transport of a Fluorescent Analogue of Glucose (2-NBDG) versus Radiolabeled Sugars by Rumen Bacteria and Escherichia coli. Biochemistry (Mosc). 2016 Maj;55(18):2578–89. 10.1021/acs.biochem.5b01286 27096355

[122] Yoshioka K, Takahashi H, Homma T, Saito M, Oh K-B, Nemoto Y, et al A novel fluorescent derivative of glucose applicable to the assessment of glucose uptake activity of Escherichia coli. Biochim Biophys Acta BBA—Gen Subj. 1996 Luty;1289(1):5–9. 10.1016/0304-4165(95)00153-0 8605231

