# Supplementary material for: Low RNA Polymerase III activity results in up regulation of HXT2 glucose transporter independently of glucose signaling and despite changing environment
Source: PLoS One. 2017 Sep 29;12(9):e0185516. doi: 10.1371/journal.pone.0185516 (PMC5621690; doi:10.1371/journal.pone.0185516)
Supplement: S1 Table — (DOCX) [file pone.0185516.s001.docx]

| Primer | Sequence |
| --- | --- |
| MAF1 270 | 5’ GCCTCCACGAGTTGCTTGTC 3’ |
| MAF230R | 5’ CCGTATTCGAAAGAAACTAAGAGC 3’ |
| MTH1_F2 | 5’ GTTTACCAAAGATTAGGATTCCATTGGCAGCCGGATTCAGTATCCCGGATCCCCGGGTTAATTAA 3’ |
| MTH1_R1 | 5’ AAGAGTCTCCAAAAAAACCATCGGGAAGGTTTCTTTTTAGTATCTGAATTCGAGCTCGTTTAAAC 3’ |
| MTH1_F | 5’ ACCTTGATGAATTGATGATGAGAT 3’ |
| Kan_R | 5’ GCGCATCGGGCTTCCCATAC 3’ |
| TUP1_F2 | 5’ GATTGTAAAGCAAGGAATTTGGAAGTATAAAAAAATAGCGCCAAATCGGATCCCCGGGTTAATTAA 3’ |
| TUP1_R1 | 5’ TAGTTAGTTACATTTGTAAAGTGTTCCTTTTGTGTTCTGTTCGAATTCGAGCTCGTTTAAAC 3’ |
| TUP1_F | 5’ CCAAAGATCGTGGTGTCCTG 3’ |
| RGT1_F2 | 5’ ATCGGCTGGAAGCTGTTGGACGATTCCGAGTTAGGCTGGTATCGGATCCCCGGGTTAATTAA 3’ |
| RGT1_R1 | 5’ AGGGAGAACCTGACCTACAGGAGAAGGGAGCATAGTTACCTGGAATTCGAGCTCGTTTAAAC 3’ |
| RGT1_F | 5’ TCCGCGAGTCATCAGTCATG 3’ |
| MIG1_F2 | 5’ CCACCCATAAGAAGTTTACCGTTGCCCTTCCCACACATGGACCGGATCCCCGGGTTAATTAA 3’ |
| MIG1_R1 | 5’ TGTCTTTTGATTTATCTGCACCGCCAAAAACTTGTCAGCGTAGAATTCGAGCTCGTTTAAAC 3’ |
| MIG1_F | 5’ GATGGCTTCCAGTAGTTCGT 3’ |
