# Supplementary material for: Low RNA Polymerase III activity results in up regulation of HXT2 glucose transporter independently of glucose signaling and despite changing environment
Source: PLoS One. 2017 Sep 29;12(9):e0185516. doi: 10.1371/journal.pone.0185516 (PMC5621690; doi:10.1371/journal.pone.0185516)
Supplement: S2 Table — (DOCX) [file pone.0185516.s002.docx]

| Plasmid | Purpose | Reference/ source |
| --- | --- | --- |
| I-0306 | *MAF1* deletion | This study |
| FA6a-3HA-kanMX6 | Protein HA-tagging | [60] |
