# Supplementary material for: Low RNA Polymerase III activity results in up regulation of HXT2 glucose transporter independently of glucose signaling and despite changing environment
Source: PLoS One. 2017 Sep 29;12(9):e0185516. doi: 10.1371/journal.pone.0185516 (PMC5621690; doi:10.1371/journal.pone.0185516)
Supplement: S3 Table — (DOCX) [file pone.0185516.s003.docx]

| Gene | Primers | Sequence |
| --- | --- | --- |
| *HXT2* | oSCZ9 (F) | 5' TGCCGAATCCTATCCTTTGC 3' |
|  | oSCZ10 (R) | 5' ACCAAACAGCCCATGAAGAC 3' |
| *HXT6/7* | HXT67FP2 (F) | 5' TCACAAGACGCTGCTATTGC 3' |
|  | HXT67RP2 (R) | 5' ACGACAGGTTCGTGCTCTTC 3' |
| *QCR9* | QCR9FP | 5’ ATCTTTGCAGGTGCCTTTGT 3’ |
|  | QCR9RP | 5’ GCAGCTATTCGAGCCTTGAC 3’ |
| *PGK* | PGK1SLL | 5’ AATCGGTGACTCCATCTTCG 3’ |
|  | PGK1SLP | 5’ CAGTGACAGTCTTGGTGTTG 3’ |
| *SCR1* | SCR1VLL | 5' GTGAGGAATCCGTCTCTCTG 3' |
|  | SCR1VLP | 5' CCGACTGATATGTGCTATCC 3' |
| *U2* | U2JCL | 5' AAGAGAAGAGCCATGACTGC 3' |
|  | U2JCP | 5' AACAGGCGTCAACCATCAAG 3' |
