# Supplementary figures and images for: Low RNA Polymerase III activity results in up regulation of HXT2 glucose transporter independently of glucose signaling and despite changing environment
Source: PLoS One. 2017 Sep 29;12(9):e0185516. doi: 10.1371/journal.pone.0185516 (PMC5621690; doi:10.1371/journal.pone.0185516)

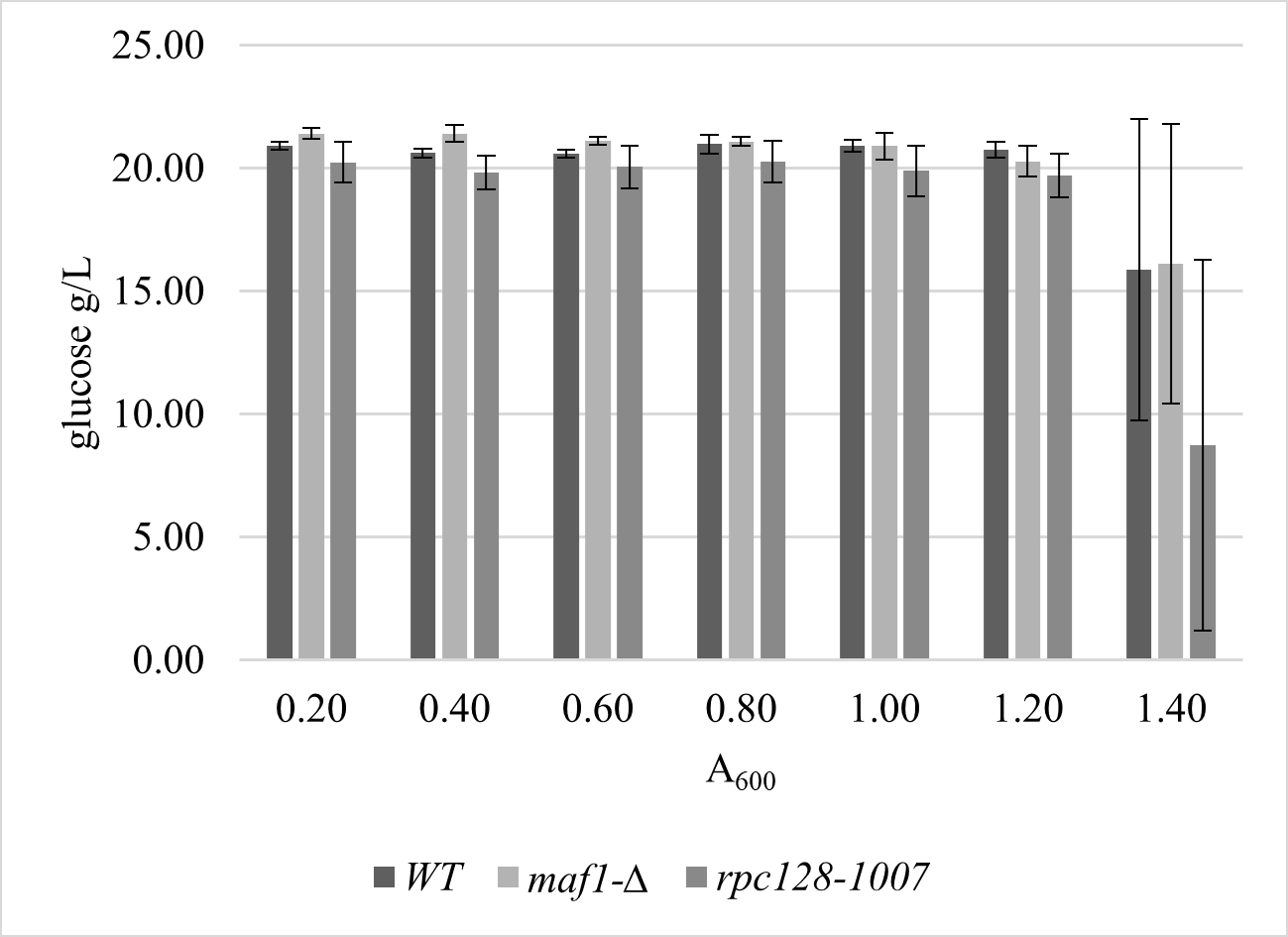

Supplement: S1 Fig — To determine the glucose concentration in media, aliquots were collected from cultures at regular intervals starting A600 = 0.2 and grown to A600 = 1.4. Data are expressed as the mean concentration, in mg of glucose in 1 ml of medium, obtained from at least three independent experiments conducted in triplicate. The standard deviation is expressed in mg of glucose in 1 ml of medium. (TIF) [file pone.0185516.s004.tif]

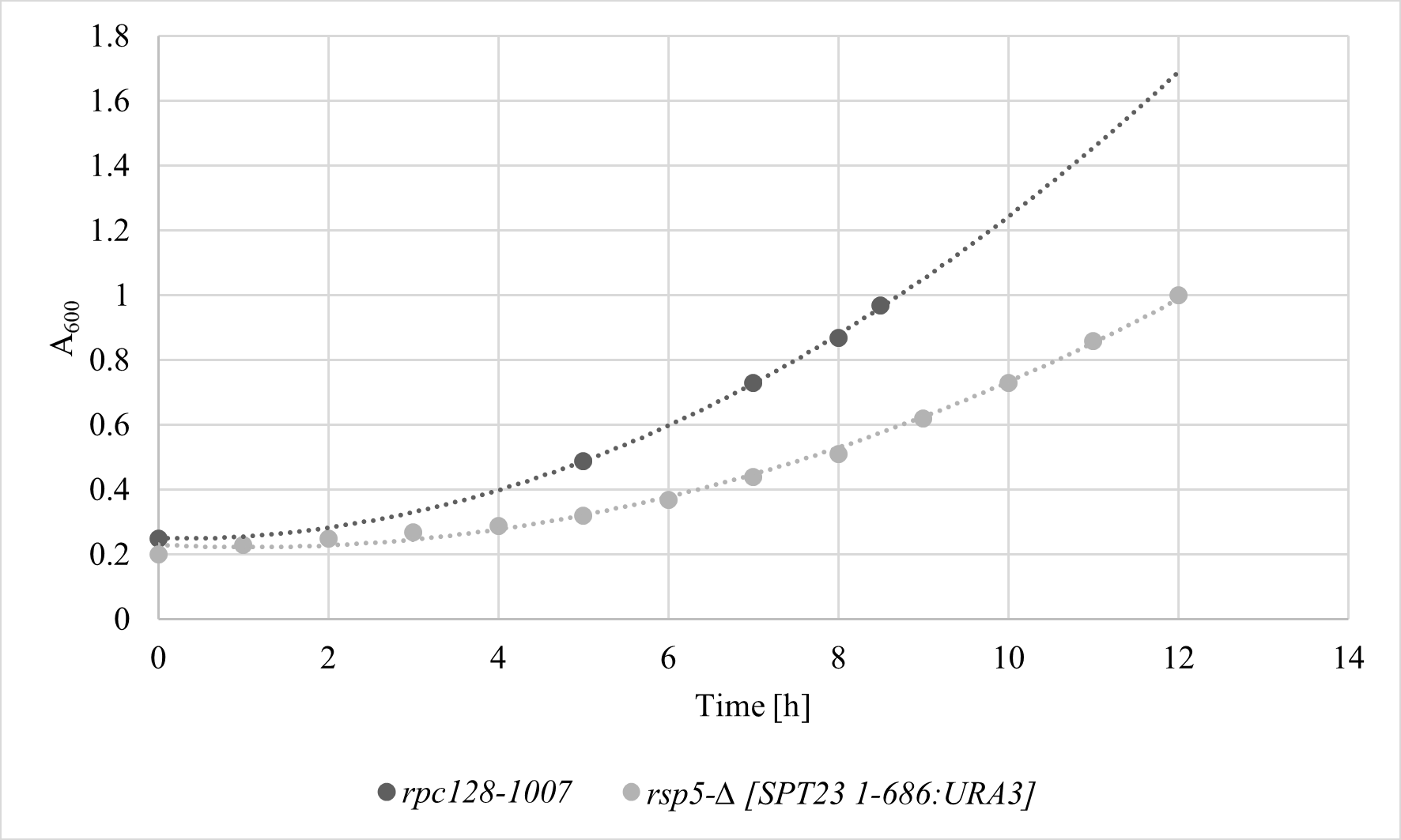

Supplement: S2 Fig — Overnight yeast cultures grown in YPD medium supplemented with 2% glucose were diluted to A600 ≈ 0.2 and cultured for 12 h. (TIF) [file pone.0185516.s005.tif]

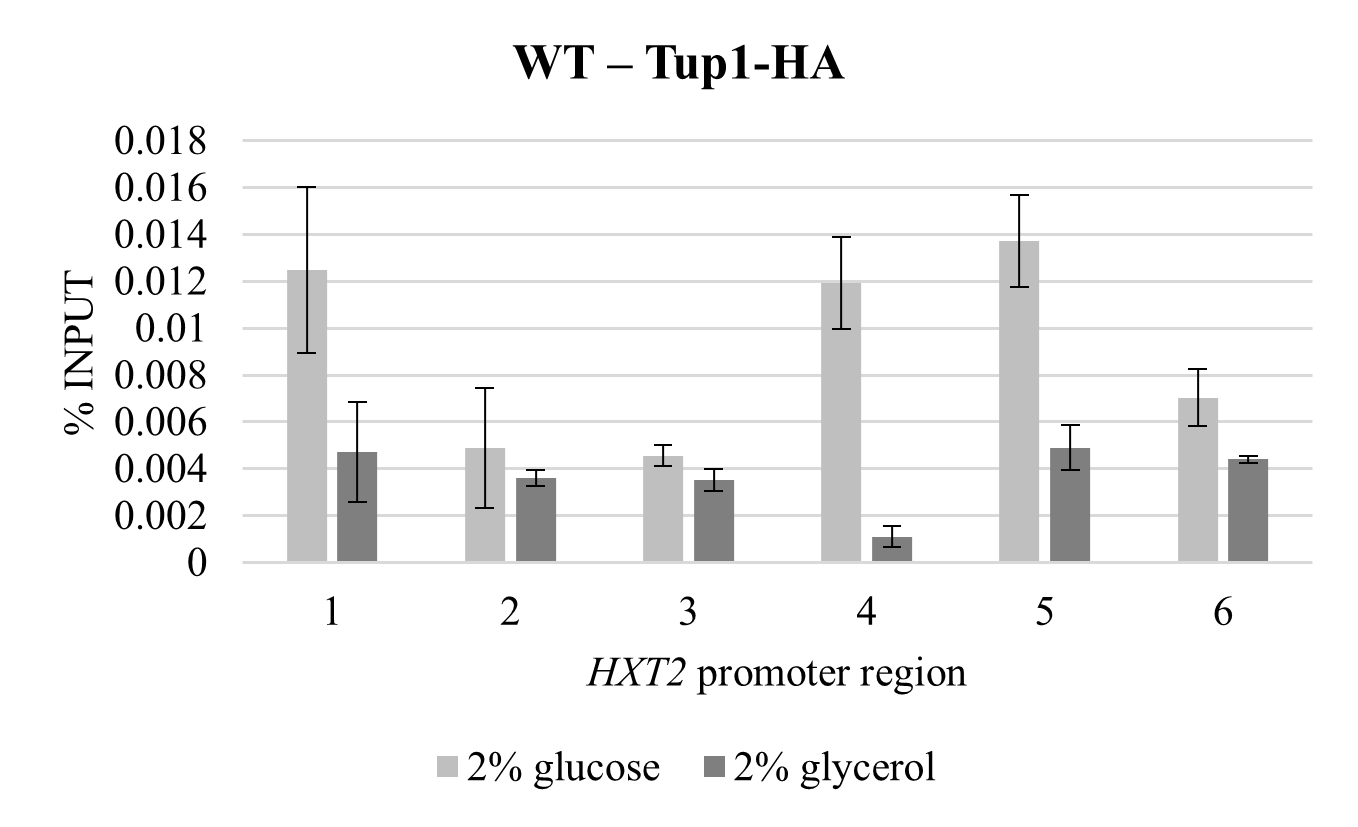

Supplement: S3 Fig — The WT (MB159-4D) strain expressing HA-tagged Tup1 was grown in 2% glucose (YPD) or 2% glycerol (YPGly) rich medium. Crosslinked chromatin was immunoprecipitated with antibodies against the HA epitope, followed by real-time PCR. The signals are presented as the percent of the INPUT signal from three separate experiments with standard deviations. Numbers from 1 to 6 correspond to real-time PCR amplification products in the HXT2 promoter region on the Fig 4A panel. (TIF) [file pone.0185516.s006.tif]

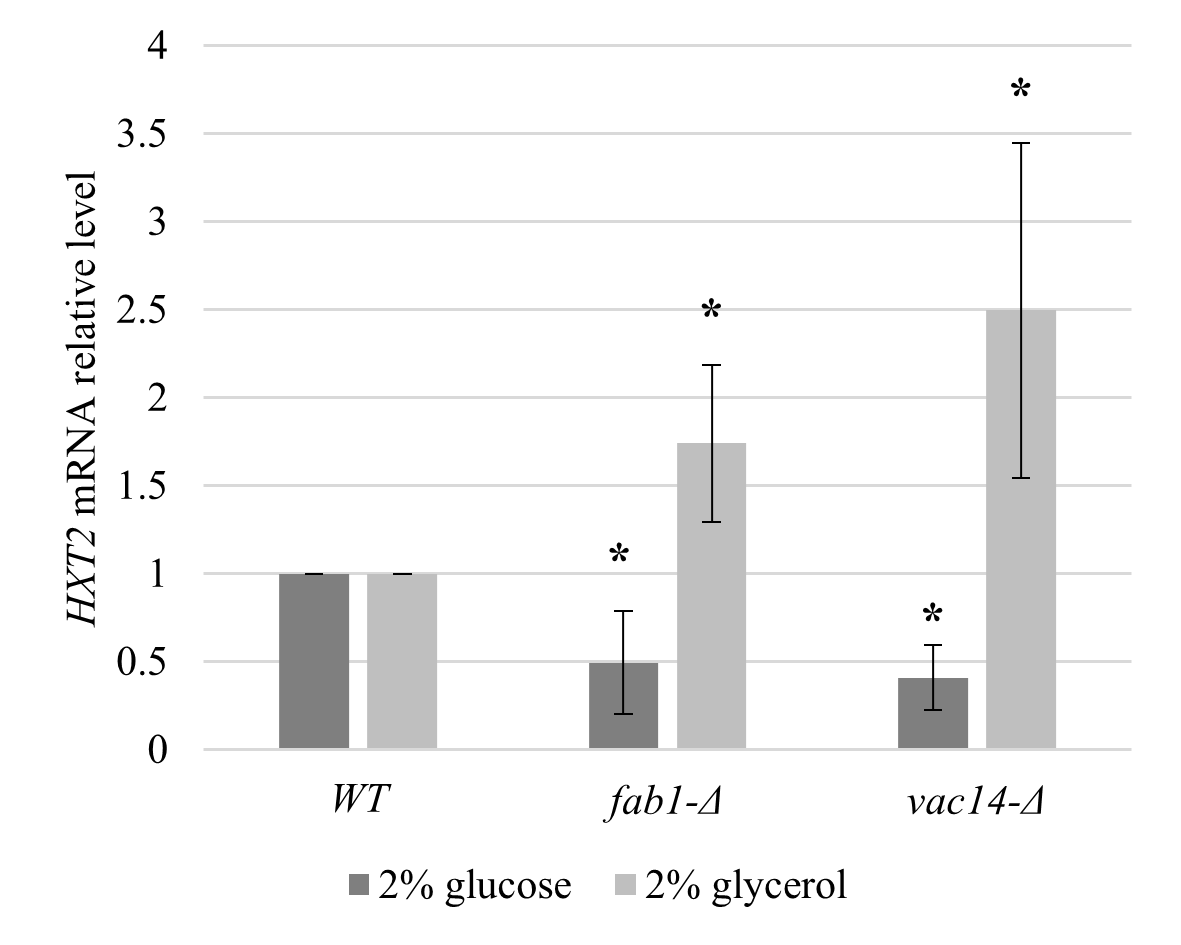

Supplement: S4 Fig — Strains were cultured in rich medium (YP) supplemented with either 2% glucose or 2% glycerol. RNA was isolated when the culture reached A600 ≈ 1 and reversed-transcribed to cDNA. Isolated RNAs were examined by SYBR GREEN-based real-time PCR. The samples were normalized to two reference genes: U2 spliceosomal RNA (U2) and small cytosolic RNA (SCR1). The bars represent ratios between the levels of respective mRNAs in the mutants and the control isogenic wild-type strain. The expression level in the WT strain (BY4741) was set as 1.0. The means ± standard deviations of the relative expression levels are shown. Asterisks (*) indicate p-values ≤ 0.05 determined by Chi Square Test. (TIF) [file pone.0185516.s007.tif]
